# An efficient synthesis of the guaiane sesquiterpene (−)-isoguaiene by domino metathesis

**DOI:** 10.3762/bjoc.15.83

**Published:** 2019-04-09

**Authors:** Yuzhou Wang, Ahmed F Darweesh, Patrick Zimdars, Peter Metz

**Affiliations:** 1 Fakultät Chemie und Lebensmittelchemie, Organische Chemie I, Technische Universität Dresden, Bergstrasse 66, 01069 Dresden, Germany,; 2 Chemistry Department, Faculty of Science, Cairo University, Giza, Egypt

**Keywords:** domino reactions, metathesis, Michael addition, organocatalysis, terpenes

## Abstract

(−)-Isoguaiene was prepared from (*S*)-citronellal in only 9–10 steps with good overall yields. Either a trienyne or a dienediyne metathesis and highly diastereoselective organocatalytic Michael additions of aldehydes derived from (*S*)-citronellal served as the key transformations.

## Introduction

**Figure 1:**
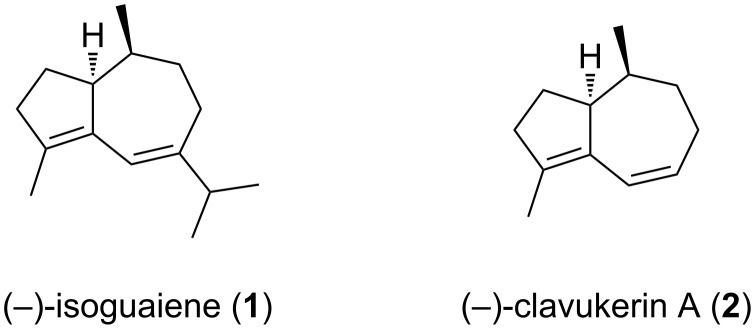
Structures of the sesquiterpene (−)-isoguaiene (**1**) and the trisnorsesquiterpene clavukerin A (**2**).

The guaiane sesquiterpene (−)-isoguaiene (**1**) has been isolated from the liverworts *Pellia epiphylla* [1] and *Dumortiera hirsuta* [2] as well as from several *Pimpinella* species [3–4], while the (+)-enantiomer of **1** has been isolated from the roots of *Parthenium hysterophorus* [5]. A recent enantioselective synthesis of (−)-isoguaiene (**1**) from (+)-dihydrocarvone [6] enabled an unambiguous assignment of its absolute configuration as depicted in Figure 1. Due to the structural similarity of **1** and the trisnorsesquiterpene clavukerin A (**2**), we were interested in developing an efficient synthetic access to **1** using a combined organocatalytic/metal-catalyzed strategy related to the one applied to the preparation of **2** [7–8].

## Results and Discussion

**Scheme 1:**
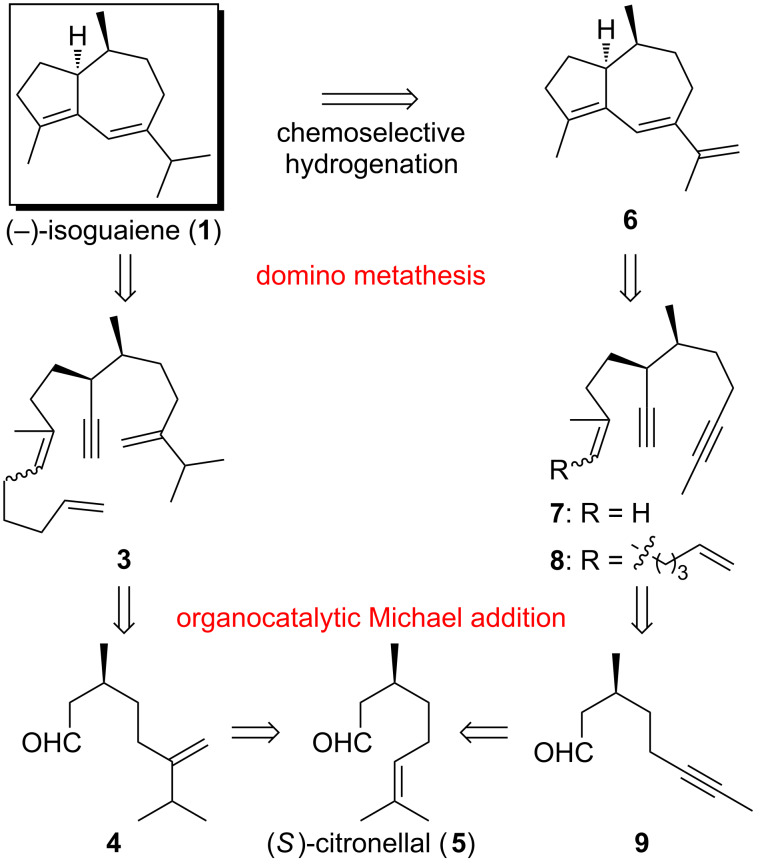
Retrosynthetic analysis for (−)-isoguaiene (**1**).

As illustrated in Scheme 1, two alternative routes were retrosynthetically devised, both of which feature a domino metathesis event and an organocatalytic Michael addition as the key steps. In closer analogy to our improved synthesis of clavukerin A (**2**) [8], a relay metathesis [9] of trienyne **3** was expected to lead to the hydroazulene **1** selectively. Trienyne **3** was envisioned to result from a stereoselective Michael addition of aldehyde **4** to methyl vinyl ketone [7–8,10] followed by chemoselective elaboration of the two carbonyl functions. Finally, aldehyde **4** was traced back to the commercially available starting material (*S*)-citronellal (**5**). On the other hand, a more rarely used enediyne metathesis [11–14] of compound **7** or its relay surrogate **8** might give rise to the conjugated triene **6**, chemoselective hydrogenation [15] of which would generate the target molecule **1**. Similar to the disconnection of trienyne **3**, the metathesis substrates **7** and **8** can be derived from aldehyde **9**, which is finally also traced back to (*S*)-citronellal (**5**).

**Scheme 2:**
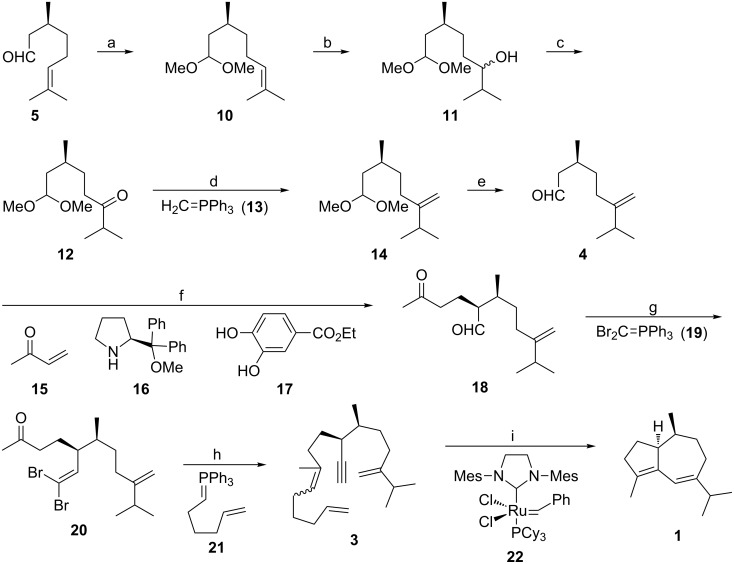
Synthesis of **1** by relay metathesis of trienyne **3**. a) HC(OMe)_3_, 4 mol % LiBF_4_, MeOH, reflux, 80%; b) (i) BH_3_·Me_2_S, THF, 0 °C to rt, (ii) 30% H_2_O_2_, 10% NaOH, 0 °C to rt, 97%; c) 5 mol % TPAP, NMO, CH_2_Cl_2_, rt, 97%; d) **13**, THF, rt to reflux, 96%; e) 25 mol % TsOH, THF, H_2_O, iPrOH, reflux, 92%; f) **15**, 7.5 mol % **16**, 20 mol % **17**, 1 °C, 91%; g) **19**, THF, 0 °C to rt, 84%; h) (i) **21**, THF, −60°C to rt then 50 °C, (ii) BuLi, −78 °C, 76%; i) 30 mol % **22**, benzene, reflux, 51%. TPAP = tetrapropylammonium perruthenate. NMO = *N*-methylmorpholine-*N*-oxide. Ts = *p*-toluenesulfonyl.

Scheme 2 illustrates the synthesis of (−)-isoguaiene (**1**) by relay metathesis of trienyne **3**. The unsaturated aldehyde **4** required for the organocatalytic Michael addition was readily prepared in five steps commencing with (*S*)-citronellal (**5**). After protection of the aldehyde function as the dimethyl acetal [16–18], hydroboration and oxidative work-up of **10** provided a mixture of epimeric alcohols **11** that was unified by Ley–Griffith oxidation [19] to give ketone **12** [20]. Subsequent Wittig reaction with ylide **13** and acetal cleavage of the resultant olefin **14** delivered aldehyde **4** with considerably higher efficiency compared to the known six-step preparation of *ent*-**4** from (−)-menthone [21]. Asymmetric Michael addition [7–8,10] of aldehyde **4** to methyl vinyl ketone (**15**) proceeded with high catalyst-controlled diastereoselectivity (dr = 23:1) to yield keto aldehyde **18**. Chemoselective dibromoolefination with ylide **19** prepared from dibromomethyltriphenylphosphonium bromide and sodium *tert*-butoxide [22] led to ketone **20** virtually without erosion of the relative configuration (dr = 22:1). After subjecting **20** to carbonyl olefination with unsaturated ylide **21** [8] followed by alkyne generation [23] with butyllithium in a one-pot process, trienyne **3** was obtained as a 1.6:1 mixture of *E* and *Z* olefin isomers. Due to the presence of the isopropyl group at the disubstituted alkene [24] of **3**, 30 mol % of the second generation Grubbs catalyst **22** were required to effect the relay metathesis of **3** to (−)-isoguaiene (**1**) in refluxing benzene in a good yield of 51%.

**Scheme 3:**
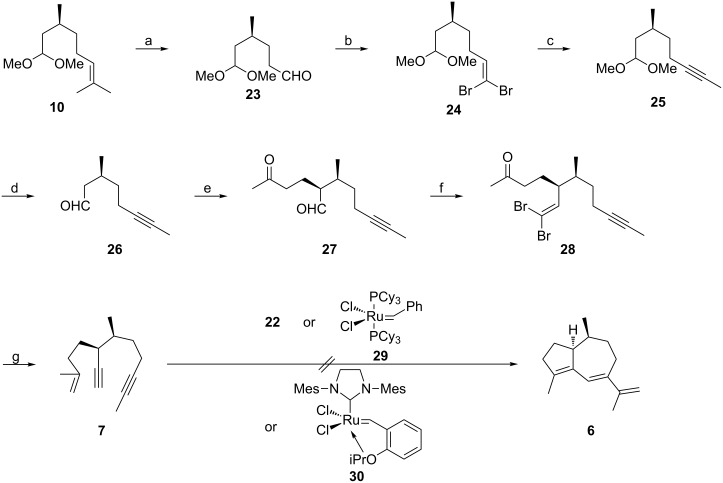
Attempted preparation of **1** by domino metathesis of enediyne **7**. a) (i) O_3_, CH_2_Cl_2_, MeOH, pyridine, −78 °C, (ii) PPh_3_, rt, 94%; b) **19**, THF, 0 °C; c) (i) BuLi, −78 °C, (ii) MeI, −78 °C to rt, 92% (2 steps); d) 25 mol % TsOH, THF, H_2_O, iPrOH, reflux, 90%; e) **15**, 10 mol % **16**, 20 mol % **17**, 2 °C; f) **19**, THF, 0 °C to rt, 85% (2 steps); g) (i) **13**, THF, rt to reflux, (ii) BuLi, −78 °C, 90%.

Thus, by application of a domino metathesis strategy featuring trienyne **3**, only 9 steps were needed to secure the guaiane sesquiterpene **1** in 19.7% overall yield starting from (*S*)-citronellal (**5**), which compares favorably with the previous synthesis of **1** from (+)-dihydrocarvone (10 steps, 6.9% overall yield) [6]. Scheme 3 depicts our first attempts to realize an alternative domino metathesis strategy using enediyne **7**.

**Scheme 4:**
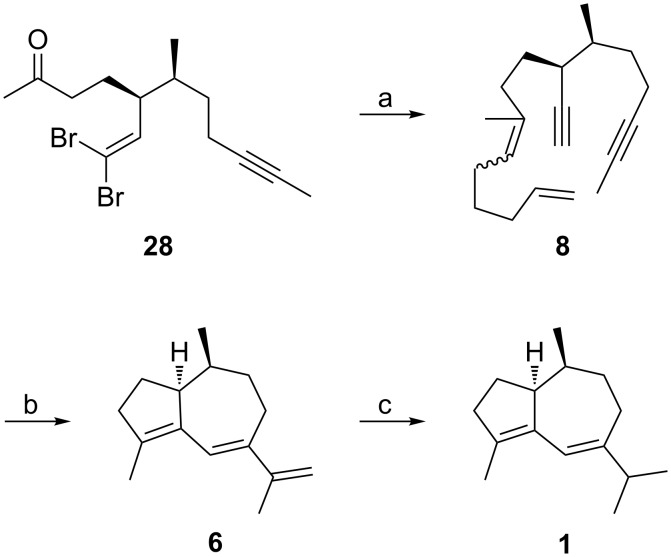
Conversion of **28** to **1** by relay metathesis of dienediyne **8**. a) (i) **21**, THF, rt to reflux, (ii) BuLi, −78 °C, 65%; b) 25 mol % **29**, CH_2_Cl_2_, reflux; c) H_2_, 15 mol % (PPh_3_)_3_RhCl, benzene, EtOH, rt, 42% (2 steps).

Ozonolysis of the unsaturated acetal **10** gave aldehyde **23** [17–18] that was subjected to dibromoolefination with ylide **19** as described for the transformation of aldehyde **18**. Use of the preformed ylide **19** led to reproducibly higher yields of **24** in comparison with the application of tetrabromomethane and triphenylphosphine [23]. One-pot alkyne formation and methylation [23] of **24** to furnish **25** and subsequent acetal hydrolysis provided the known aldehyde **26** [25] in very good overall yield. In our hands, the "demethanation" of (*S*)-citronellol to produce the primary alcohol corresponding to aldehyde **26** according to the protocol of Abidi (NaNO_2_, aqueous AcOH) [26] as a potential shortcut to **26** only proceeded with a maximum yield of 20%. Asymmetric Michael addition of aldehyde **26** to methyl vinyl ketone (**15**) followed immediately by treatment of the resultant unstable keto aldehyde **27** with ylide **19** delivered dibromo olefin **28** with high diastereocontrol (dr = 19:1). Olefination of ketone **28** with ylide **13** and alkyne formation with butyllithium in a one-pot procedure then gave rise to enediyne **7** in excellent yield. Unfortunately, all attempts to achieve a domino metathesis of **7** to hydroazulene **6** only met with failure. Thus, neither the Grubbs catalysts **22** or **29**, nor the Hoveyda–Blechert catalyst **30** [27–28] in the presence or absence of ethylene effected the desired transformation to triene **6**. As a consequence, we resorted to a relay strategy for enediyne metathesis as well, and the successful execution of this idea is illustrated in Scheme 4.

Similar to the transformation of ketone **20**, the one-pot conversion of ketone **28** by olefination with unsaturated ylide **21** and alkyne formation with butyllithium yielded dienediyne **8** as a 1.3:1 mixture of *E* and *Z* olefin isomers. Gratifyingly, treatment of **8** with 25 mol% of the first generation Grubbs catalyst **29** produced the desired hydroazulene **6** in refluxing dichloromethane. Without purification, the crude sensitive conjugated triene **6** was immediately hydrogenated in the presence of the Wilkinson catalyst [29] to give (−)-isoguaiene (**1**) by chemoselective reduction of only the terminal olefin [15] in satisfactory yield over the 2 steps. Hence, the natural product **1** was available through this domino metathesis strategy featuring dienediyne **8** in 10 steps from (*S*)-citronellal (**5**) in 14.5% overall yield.

## Conclusion

In summary, we have accomplished two short and efficient catalytic routes from (*S*)-citronellal (**5**) to the guaiane sesquiterpene (−)-isoguaiene (**1**) using either a trienyne or a dienediyne metathesis and highly diastereoselective organocatalytic Michael additions of aldehydes derived from **5** as the key steps.

## Supporting Information

File 1 Experimental procedures and copies of ^1^H NMR and ^13^C NMR spectra of compounds **1**, **3**, **4**, **7**, **8**, **10**, **12**, **14**, **18**, **20**, **23**–**28**.
