# Supplementary material for: An efficient synthesis of the guaiane sesquiterpene (−)-isoguaiene by domino metathesis
Source: Beilstein J Org Chem. 2019 Apr 9;15:858–62. doi: 10.3762/bjoc.15.83 (PMC6466796; doi:10.3762/bjoc.15.83)

BEILSTEIN JOURNAL OF ORGANIC CHEMISTRY

Supporting Information

for

An efficient synthesis of the guaiane sesquiterpene (−)-isoguaiene by domino metathesis

Yuzhou Wang, Ahmed F. Darweesh, Patrick Zimdars and Peter Metz

Beilstein J. Org. Chem. **2019,** *15,* 858–862. doi:10.3762/bjoc.15.83

Experimental procedures and copies of ^{1}H NMR and ^{13}C NMR spectra of compounds 1, 3, 4, 7, 8, 10, 12, 14, 18, 20, 23–28

License and Terms: This is a supporting information file under the terms of the Creative Commons Attribution License (http://creativecommons.org/licenses/by/4.0). Please note that the reuse, redistribution and reproduction in particular requires that the authors and source are credited.
The license is subject to the *Beilstein Journal of Organic Chemistry* terms and conditions: (https://www.beilstein-journals.org/bjoc)

General information: THF, CH_2Cl_2, and EtOH were dried and purified by passage through a MB-SPS-800 device using molecular sieves. All other commercially available reagents were used as received. Reactions were performed under argon atmosphere. Thin layer chromatography was performed on Merck silica gel 60 F_{254} 0.2 mm precoated plates. Product spots were visualized by UV light at 254 nm and subsequently developed using anisaldehyde solution as appropriate. Flash column chromatography was carried out using silica gel (Merck, particle size 40–63 microns). Melting points were measured on a Wagner & Munz PolyTherm A and are uncorrected. Infrared spectra were recorded on a THERMONICOLET Avatar 360 instrument using ATR. NMR spectra were recorded on a Bruker AC 300 P (300 MHz ^{1}H, 75 MHz ^{13}C), on a Bruker DRX 500 P (500 MHz ^{1}H, 125 MHz ^{13}C) or on a Bruker AC 600-P (600 MHz ^{1}H, 151 MHz ^{13}C) spectrometer. Chemical shifts (δ) are quoted in parts per million (ppm) downfield of tetramethylsilane, using residual proton-containing solvent as internal standard ($CDCl_3$ at 7.26 ppm). Abbreviations used in the description of resonances are: s (singlet), d (doublet), t (triplet), q (quartet), br (broad). Coupling constants (*J*) are quoted to the nearest 0.1 Hz. Mass spectra were recorded with an Agilent 5973N detector coupled with an Agilent 6890N GC (GC–MS, 70 eV) or else with a Bruker Esquire-LC (direct injection as a methanolic NH_4OAc solution, ESI). HRMS spectra were recorded on a Bruker Daltonics "Impact II" (ESI–TOF). Elemental analysis was performed on a Hekatech EA 3000.

Dimethylacetal 10: A solution of (*S*)-citronellal (**5**, 2.26 g, 14.7 mmol), $LiBF_4$ (65.2 mg, 0.695 mmol), and $HC(OMe)_3$ (2.3 mL, 21 mmol) in methanol was heated at reflux for 22 h. After letting the mixture cool to room temperature, a saturated aqueous solution of $NaHCO_3$ was added. The mixture was extracted with ethyl acetate, the combined organic

layers were washed with brine and dried over $MgSO_4$, and the solvent was removed under reduced pressure. Purification of the residue by flash chromatography (isohexane/ethyl acetate 30:1) afforded dimethyl acetal **10** (2.36 g, 80%) as a colorless oil.

10: R_f = 0.43 (isohexane/ethyl acetate, 20:1); $[\alpha]_D^{24}$ = –6.7 (*c* 1.13, $CHCl_3$); ^1H NMR (500 MHz, $CDCl_3$) δ 5.13–5.05 (m, 1H), 4.46 (dd, *J* = 6.6, 5.0 Hz, 1H), 3.32 (s, 3H), 3.30 (s, 3H), 2.06–1.90 (m, 2H), 1.68 (d, *J* = 0.9 Hz, 3H), 1.67–1.61 (m, 1H), 1.60 (s, 3H), 1.59–1.52 (m, 1H), 1.40–1.30 (m, 2H), 1.22–1.14 (m, 1H), 0.92 (d, *J* = 6.6 Hz, 3H) ppm; ^{13}C NMR (125 MHz, $CDCl_3$) δ 131.4 (C), 124.8 (CH), 103.3 (CH), 52.9 (CH_3), 52.3 (CH_3), 39.6 (CH_2), 37.5 (CH_2), 28.9 (CH), 25.9 (CH_3), 25.5 (CH_2), 19.8 (CH_3), 17.8 (CH_3) ppm; IR (ATR) ν: 2917, 2850, 2826, 1455, 1377, 1193, 1124, 1053, 965, 913, 830, 741 cm^{-1}; GC–MS: *m/z* = 169 $[M–OMe]^+$; anal. calcd for $C_{12}H_{24}O_2$: C 71.95, H 12.08; found: C 71.92, H 12.28.

Alcohols 11: To a solution of dimethylacetal **10** (1.30 g, 6.50 mmol) in THF (35 mL) cooled to 0 °C was added dropwise a solution of $BH_3·Me_2S$ in THF (2 M, 4.87 mL, 9.75 mmol), and stirring was continued at room temperature for 18 h. Then a 10% aqueous solution of NaOH (5.5 mL) and a 30% aqueous solution of H_2O_2 (3.25 mL) were added, and stirring was continued for 6 h at room temperature. The reaction mixture was diluted with a half-saturated aqueous solution of NaCl (20 mL) and extracted 3× with a mixture of dichloromethane and pentane (4:6, 20 mL). The combined organic layers were washed with a saturated aqueous solution of $Na_2S_2O_3$ (10 mL) and dried over $MgSO_4$. After removal of the solvents at reduced pressure, purification of the residue by flash chromatography (dichloromethane/diethyl ether, 9:1 and 7:3) gave the epimeric alcohols **11** (1.38 g, 97%) as a colorless oil that was converted to ketone **12** without full

characterization. From another run, a sample of alcohols **11** was analyzed by mass spectrometry.

11: R_f = 0.34 (dichloromethane/diethyl ether, 7:3); MS (ESI) *m/z*: 236.2 $[M+NH_4]^+$.

Ketone 12: $MgSO_4$ (3.03 g, 25.4 mmol) was added to a solution of NMO monohydrate (3.41 g, 25.4 mmol) in dichloromethane (40 mL), and the mixture was stirred for 1 h at room temperature. Subsequently, this NMO solution was transferred to a solution of the epimeric alcohols **11** (1.38 g, 6.32 mmol) in dichloromethane (20 mL) by syringe through a cotton plug followed by TPAP (0.111 g, 0.316 mmol), and stirring was continued for 1.5 h at room temperature. Then the mixture was filtered through a pad of silica gel (elution with dichloromethane/diethyl ether, 7:3), and concentrated under reduced pressure. Flash chromatography over silica gel (dichloromethane/diethyl ether, 15:1) afforded ketone **12** (1.32 g, 97% over 2 steps) as a colorless oil.

12: R_f = 0.33 (dichloromethane/diethyl ether, 15:1); $[\alpha]_D^{25}$ = –5.4 (*c* 2.21, CH_2Cl_2); ^1H NMR (300 MHz, $CDCl_3$) δ 4.43 (dd, *J* = 6.4, 5.1 Hz, 1H), 3.29 (s, 3H), 3.28 (s, 3H), 2.58 (dt, *J* = 13.8, 7.0 Hz, 1H), 2.50–2.36 (m, 2H), 1.67–1.47 (m, 3H), 1.47–1.30 (m, 2H), 1.06 (d, *J* = 7.0 Hz, 6H), 0.90 (d, *J* = 6.4, Hz, 3H) ppm; ^{13}C NMR (75 MHz, $CDCl_3$) δ 214.77 (C), 103.0 (CH), 52.7 (CH_3), 52.4 (CH_3), 40.8 (CH), 39.3 (CH_2), 37.8 (CH_2), 30.7 (CH_2), 28.7 (CH), 19.6 (CH_3), 18.27 (CH_3), 18.24 (CH_3) ppm; IR (ATR) ν: 2935, 2830, 1710, 1685, 1652, 1462, 1380, 1124, 1051, 960 cm^{-1}; MS (ESI) *m/z*: 234.2 $[M+NH_4]^+$, 217.1 $[M+H]^+$.

Olefin 14: To a suspension of methyltriphenylphosphonium bromide (5.63 g, 15.8 mmol) in THF (48 mL) was slowly added a solution of BuLi in hexane (1.5 M, 10.2 mL, 15.3

mmol) at 0° C. The mixture was stirred for 10 min at 0 °C and for 1 h at room temperature, a solution of ketone **12** (1.10 g, 5.09 mmol) in THF (10 mL) was added, and the resulting mixture was stirred at room temperature for 1 h and then heated to reflux for 2.5 h. After allowing the mixture to cool to room temperature, isohexane (60 mL) was added, the mixture was filtered through a plug of silica gel with diethyl ether, and the solvents were removed under reduced pressure. Purification of the residue by flash chromatography (pentane/diethyl ether, 20:1) gave olefin **14** (1.05 g, 96%) as a colorless oil.

14: R_f = 0.26 (dichloromethane/diethyl ether, 20:1); $[\alpha]_D^{25}$ = –6.5 (*c* 1.2, CH_2Cl_2); ^{1}H NMR (300 MHz, $CDCl_3$) δ 4.72 (br s, 1H), 4.68–4.65 (m, 1H), 4.47 (dd, *J* = 6.3, 5.2 Hz, 1H), 3.31 (s, 3H), 3.30 (s, 3H), 2.31–2.15 (m, 1H), 2.03 (td, *J* = 10.1, 5.7 Hz, 2H), 1.72–1.53 (m, 2H), 1.53–1.33 (m, 2H), 1.33–1.21 (m, 1H), 1.02 (d, *J* = 6.8 Hz, 6H), 0.93 (d, *J* = 6.4 Hz, 3H) ppm; ^{13}C NMR (75 MHz, $CDCl_3$) δ 156.2 (C), 106.1 (CH_2), 103.1 (CH), 52.7 (CH_3), 52.2 (CH_3), 39.5 (CH_2), 35.8 (CH_2), 33.7 (CH), 31.6 (CH_2), 29.0 (CH), 21.90 (CH_3), 21.84 (CH_3), 19.8 (CH_3) ppm; IR (ATR) ν: 2957, 2926, 2872, 2829, 1641, 1459, 1378, 1192, 1125, 1054, 962, 887 cm^{-1}; GC–MS: *m/z* = 182 $[M–MeOH]^+$; anal. calcd for $C_{13}H_{26}O_2$: C 72.85, H 12.23; found: C 72.65, H 12.59.

Aldehyde 4: A mixture of dimethylacetal **14** (1.13 g, 5.27 mmol), TsOH·H_2O (250 mg, 1.32 mmol), water (9 mL), isopropanol (1.2 mL), and THF (18 mL) was heated for 90 min under reflux. After letting the mixture cool to room temperature, isohexane (20 mL) was added, and the mixture was extracted with diethyl ether (3 × 10 mL). The combined organic layers were washed with brine (10 mL) and dried over $MgSO_4$. After filtration through a plug of silica gel, the solvents were removed under reduced pressure to give the instable

aldehyde **4** (817 mg, 92%) as a colorless oil that was rapidly converted to keto aldehyde **18**.

4: R_f = 0.28 (pentane/diethyl ether, 20:1); $[\alpha]_D^{25}$ = –17.0 (*c* 1.27, CH_2Cl_2); ^{1}H NMR (300 MHz, $CDCl_3$) δ 9.75 (t, *J* = 2.3 Hz, 1H), 4.74 (s, 1H), 4.68–4.64 (m, 1H), 2.42 (ddd, *J* = 15.9, 5.7, 1.9 Hz, 1H), 2.30–2.16 (m, 2H), 2.14–1.94 (m, 3H), 1.55–1.31 (m, 2H), 1.02 (d, *J* = 6.8 Hz, 6H), 0.98 (d, *J* = 6.6 Hz, 3H) ppm; ^{13}C NMR (75 MHz, $CDCl_3$) δ 202.8 (CH), 155.6 (C), 106.5 (CH_2), 51.0 (CH_2), 35.3 (CH_2), 33.7 (CH), 31.7 (CH_2), 28.0 (CH), 21.84 (CH_3), 21.80 (CH_3), 19.9 (CH_3) ppm; IR (ATR) ν: 3076, 2960, 2930, 2873, 2714, 1724, 1700, 1648, 1650, 1461, 1379, 1110, 887 cm^{-1}; GC–MS: *m/z* = 153 $[M–Me]^+$.

Keto aldehyde 18: In a pointed flask, a mixture of aldehyde **4** (936 mg, 5.56 mmol) and methyl vinyl ketone (**15**, 1.17 g, 16.7 mmol) cooled to 0 °C was treated successively with proline derivative **16** (112 mg, 0.417 mmol) and catechol **17** (202.6 mg, 1.112 mmol). After stirring for 3 d at 1 °C, the mixture was directly subjected to flash chromatography (pentane/diethyl ether, 4:1) to give the keto aldehyde **18** (1.205 g, 91%, dr = 23:1 according to ^{1}H NMR integration) as a colorless oil.

18: R_f = 0.21 (pentane/diethyl ether, 4:1); $[\alpha]_D^{25}$ = +30.9 (*c* 1.15, CH_2Cl_2); ^{1}H NMR (600 MHz, $CDCl_3$) δ 9.64 (d, *J* = 2.6 Hz, 1H), 4.74 (s, 1H), 4.65 (s, 1H), 2.51 (ddd, *J* = 17.8, 8.8, 5.6 Hz, 1H), 2.37 (ddd, *J* = 17.8, 8.3, 6.8 Hz, 1H), 2.23–2.17 (m, 2H), 2.13–2.11 (m, 3H), 2.14–2.06 (m, 1H), 2.00–1.93 (m, 1H), 1.93–1.85 (m, 2H), 1.75–1.68 (m, 1H), 1.58–1.51 (m, 1H), 1.38–1.30 (m, 1H), 1.01 (d, *J* = 6.8 Hz, 3H), 1.01 (d, *J* = 6.8, 3H), 1.00 (d, *J* = 7.2 Hz, 3H) ppm; ^{13}C NMR (150 MHz, $CDCl_3$) δ 208.1 (C), 205.3 (CH), 155.4 (C), 106.7 (CH_2), 56.3 (CH), 41.4 (CH_2), 33.58 (CH), 33.57 (CH), 32.3 (CH_2), 31.9 (CH_2), 30.0 (CH_3), 21.86

(CH_3), 21.77 (CH_3), 19.7 (CH_2), 17.0 (CH_3) ppm; IR (ATR) ν: 2960, 2930, 2871, 2711, 1714, 1685, 1652, 1363, 1231, 1164, 887 cm^{-1}; MS (ESI) *m/z*: 239.2 $[M+H]^+$.

Dibromo olefin 20: To a suspension of dibromomethyltriphenylphosphonium bromide (5.26 g, 10.2 mmol) in THF (50 mL) cooled to 0 °C was added sodium *tert*-butoxide (951 mg, 9.00 mmol). After stirring for 10 min at 0 °C and 20 min at room temperature, the mixture was cooled to 0 °C and treated with a solution of keto aldehyde **18** (1.18 g, 4.95 mmol) in THF (10 mL). Stirring was continued for 2 h at 0 °C and 30 min at room temperature. Pentane (50 mL) was added, and the solids were removed by filtration through a plug of silica gel. The solvents were evaporated at reduced pressure, and purification of the residue by flash chromatography (pentane/diethyl ether, 15:1) afforded dibromo olefin **20** (1.64 g, 84%, dr = 22:1 according to ^1H NMR integration) as a slightly yellow oil.

20: R_f = 0.28 (pentane/diethyl ether, 15:1); $[\alpha]_D^{25}$ = +7.1 (*c* 1.04, CH_2Cl_2); ^1H NMR (600 MHz, $CDCl_3$) δ 6.17 (d, *J* = 10.5 Hz, 1H), 4.74 (s, 1H), 4.69–4.65 (m, 1H), 2.46–2.41 (m, 2H), 2.41–2.34 (m, 1H), 2.25–2.18 (m, 1H), 2.15 (s, 3H), 2.10–1.97 (m, 2H), 1.79–1.72 (m, 1H), 1.60–1.53 (m, 2H), 1.52–1.45 (m, 1H), 1.31–1.23 (m, 1H), 1.02 (d, *J* = 6.8 Hz, 3H), 1.02 (d, *J* = 6.8 Hz, 3H), 0.89 (d, *J* = 6.8 Hz, 3H) ppm; ^{13}C NMR (75 MHz, $CDCl_3$) δ 208.3 (C), 155.9 (C), 140.3 (CH), 106.4 (CH_2), 89.2 (C), 47.5 (CH), 41.2 (CH_2), 36.7 (CH), 33.6 (CH), 33.4 (CH_2), 32.0 (CH_2), 30.1 (CH_3), 25.8 (CH_2), 21.91 (CH_3), 21.87 (CH_3), 15.7 (CH_3) ppm; IR (ATR) ν: 3075, 2960, 2929, 2872, 1715, 1640, 1456, 1362, 1248, 1162, 887 cm^{-1}; MS (ESI) *m/z*: 410.3 $[M+NH_4]^+$, 393.2 $[M+H]^+$; HRMS (ESI) *m/z*: $[M+Na]^+$ calcd for $C_{16}H_{28}Br_2NaO^+$, 415.0248; found, 415.0250.

Trienyne 3: To a suspension of 5-hexenyltriphenylphosphonium bromide (4.608 g, 10.83 mmol) in THF (55 mL) cooled to −60 °C was added dropwise a solution of BuLi in hexane (1.5 M, 6.96 mL, 10.4 mmol). The mixture was stirred at room temperature for 1.5 h and then cooled to −60 °C again. A solution of dibromo olefin **20** (1.054 g, 2.675 mmol) in THF (13 mL) was added, and the resulting mixture was stirred at room temperature for 2 d and at 50 °C for 3 h. After cooling to −78 °C, a solution of BuLi in hexane (1.4 M, 5.72 mL, 8.00 mmol) was added, and stirring was continued −78 °C for 2 h. The mixture was diluted with pentane (60 mL), allowed to warm to room temperature, and filtered through a plug of silica gel. The solvents were removed under reduced pressure, and purification of the residue by flash chromatography (pentane) gave trienyne **3** (607 mg, 76%, 1.6:1 mixture of *E* and *Z* olefin isomers) as a colorless oil.

3: R_f = 0.29 (pentane); ^{1}H NMR (300 MHz, $CDCl_3$) δ 5.90–5.74 (m, 1H), 5.17 (d, *J* = 6.2 Hz, 1H), 5.06–4.90 (m, 2H), 4.74 (br s, 1H), 4.69 (br s, 1H), 2.42–2.31 (m, 1H), 2.30–2.12 (m, 3H), 2.12–1.95 (m, 7H), 1.69 (s, 2H), 1.66–1.37 (m, 8H), 1.03 (d, *J* = 6.8 Hz, 6H), 0.96 (d, *J* = 6.4 Hz, 3H) ppm; IR (ATR) ν: 3308, 3078, 2961, 2928, 2872, 1641, 1454, 1379, 992, 909, 888, 626 cm^{-1}; GC–MS: *m/z* = 285 $[M–Me]^+$.

(−)-Isoguaiene (1) from 3: In a round-bottom 100 mL flask equipped with a Vigreux column, which was connected via a two-neck (Anschütz) attachment to a reflux condenser, a solution of catalyst **22** (71.0 mg, 0.0835 mmol) in benzene (20 mL) was heated to reflux (oil bath temperature 110 °C). A solution of trienyne **3** (250.0 mg, 0.832 mmol) in benzene (50 mL) was added through one neck of the Anschütz attachment over 4 h, and the resulting mixture was stirred for 30 h in total at reflux. Further portions of a solution of catalyst **22** (71.0 mg, 0.0835 mmol) in benzene (10 mL) were added after 2 and 22 h of

reflux. The mixture was allowed to cool to room temperature and filtered through a pad of silica gel with pentane. The solvents were carefully removed under reduced pressure, and the residue was purified by flash chromatography (pentane) to give (−)-isoguaiene **1** (86.6 mg, 51%) as a colorless oil.

1: R_f = 0.67 (pentane); $[\alpha]_D^{25}$ = –45.1 (*c* 1.13, $CHCl_3$); ^1H NMR (600 MHz, $CDCl_3$) δ 6.04 (br s, 1H), 3.08–3.02 (m, 1H), 2.33–2.26 (m, 3H), 2.25–2.17 (m, 1H), 2.06 (ddd, *J* = 17.1, 9.8, 3.2 Hz, 1H), 1.97–1.91 (m, 1H), 1.88 (dtd, *J* = 12.8, 9.0, 9.0, 5.5 Hz, 1H), 1.82–1.76 (m, 1H), 1.73 (d, *J* = 1.9 Hz, 3H), 1.60–1.52 (m, 2H), 1.02 (d, *J* = 6.8 Hz, 3H), 1.01 (d, *J* = 6.8 Hz, 3H), 0.77 (d, *J* = 7.2 Hz, 3H) ppm; ^{13}C NMR (150 MHz, $CDCl_3$) δ 147.9 (C), 136.1 (C), 134.6 (C), 118.0 (CH), 52.1 (CH), 38.5 (CH), 37.8 (CH_2), 35.4 (CH_2), 34.6 (CH), 27.03 (CH_2), 26.97 (CH_2), 21.73 (CH_3), 21.67 (CH_3), 14.4 (CH_3), 13.7 (CH_3) ppm; IR (ATR) ν: 2957, 2911, 2869, 1658, 1640, 1614, 1457, 1445, 1395, 1379 cm^{-1}; GC–MS: *m/z* = 204 $[M]^+$.

Aldehyde 23: O_3 was bubbled through a solution of dimethyl acetal **10** (985 mg, 4.92 mmol) in pyridine (2.9 mL, 36 mmol) and methanol/CH_2Cl_2 (1:1, 20 mL) for 15 min at −78 °C. Afterwards, N_2 was bubbled through the solution until the blue color disappeared. PPh_3 (10 g, 38 mmol) was added, and the reaction mixture was stirred for 3 h at room temperature. After concentrating the reaction mixture under reduced pressure, isohexane was added, and the solids were removed by filtering through a plug of silica gel. The filtrate was concentrated under reduced pressure, and purification of the residue by flash chromatography (isohexane/ethyl acetate, 10:1 to 5:1) afforded the unstable aldehyde **23** (807 mg, 94%) as a colorless oil.

23: R_f = 0.42 (isohexane/ethyl acetate, 4:1); $[\alpha]_D^{21}$ = –4.4 (*c* 0.38, $CHCl_3$); ^1H NMR (600 MHz, $CDCl_3$) δ 9.78 (t, *J* = 1.7 Hz, 1H), 4.51–4.42 (m, 1H), 3.32 (s, 3H), 3.31 (s, 3H), 2.52–2.38 (m, 2H), 1.74–1.66 (m, 1H), 1.66–1.55 (m, 2H), 1.52–1.45 (m, 1H), 1.45–1.38 (m, 1H), 0.93 (d, *J* = 6.4 Hz, 3H) ppm; ^{13}C NMR (150 MHz, $CDCl_3$) δ 202.7 (CH), 103.1 (CH), 52.9 (CH_3), 52.7 (CH_3), 41.7 (CH_2), 39.4 (CH_2), 29.1 (CH_2), 28.9 (CH), 19.7 (CH_3) ppm; GC–MS: *m/z* = 143 $[M–OMe]^+$; HRMS (ESI) *m/z*: $[M+Na]^+$ calcd for $C_9H_{18}NaO_3^+$, 197.1148; found, 197.1146.

Dibromo olefin 24: To a suspension of dibromomethyltriphenylphosphonium bromide (8.27 g, 16.1 mmol) in THF (80 mL) cooled to 0 °C was added sodium *tert*-butoxide (1.466 g, 15.26 mmol). After stirring for 10 min at 0 °C and 20 min at room temperature, the mixture was cooled to 0 °C and treated with a solution of aldehyde **23** (1.40 g, 8.03 mmol) in THF (16 mL). Stirring was continued for 1 h at 0 °C, and then the mixture was filtered through a plug of silica gel with diethyl ether. After removal of the solvents at reduced pressure, purification of the residue by flash chromatography (pentane/diethyl ether, 15:1) gave dibromo olefin **24** as an unstable liquid. From another run, a sample of dibromo olefin **24** was analyzed by ^1H NMR spectroscopy.

24: R_f = 0.26 (pentane/diethyl ether, 15:1); ^1H NMR (300 MHz, $CDCl_3$) δ 6.38 (t, *J* = 7.3 Hz, 1H), 4.46 (dd, *J* = 6.6, 5.5 Hz, 1H), 3.32 (s, 3H), 3.31 (s, 3H), 2.21–2.01 (m, 2H), 1.70–1.55 (m, 2H), 1.37–1.15 (m, 3H), 0.95 (d, *J* = 6.4 Hz, 3H) ppm.

Alkyne 25: To a solution of the dibromo olefin **24** obtained above in THF (50 mL) cooled to −78 °C was added a solution of BuLi in hexane (1.6 M, 17.6 mL, 28.1 mmol). After stirring the mixture at −78 °C for 75 min, methyl iodide (9.12 g, 62.2 mmol) was added,

and the mixture was allowed to warm to room temperature with stirring overnight. A saturated, aqueous $NaHCO_3$ solution (24 mL) was added, and the aqueous layer was extracted with isohexane (2 × 20 mL), and the combined organic layers were washed with brine and dried over $MgSO_4$. After removal of the solvents under reduced pressure, flash chromatography (pentane/diethyl ether, 15:1) of the residue yielded alkyne **25** (1.365 g, 92% over 2 steps) as a colorless oil.

25: R_f = 0.18 (pentane/diethyl ether, 15:1); $[\alpha]_D^{24}$ = –7.7 (*c* 1.0, $CHCl_3$); ^{1}H NMR (600 MHz, $CDCl_3$) δ 4.45 (dd, *J* = 6.6, 5.1 Hz, 1 H), 3.30 (s, 3 H), 3.28 (s, 3 H), 2.19–2.05 (m, 2 H), 1.75 (t, *J* = 2.6 Hz, 3 H), 1.71–1.65 (m, 1 H), 1.65–1.60 (m, 1 H), 1.55–1.47 (m, 1 H), 1.38–1.30 (m, 2 H), 0.90 (d, *J* = 6.4 Hz, 3 H) ppm; ^{13}C NMR (150 MHz, $CDCl_3$) δ 102.9 (CH), 79.1 (C), 75.4 (C), 52.8 (CH_3), 51.9 (CH_3), 39.1 (CH_2), 36.4 (CH_2), 28.3 (CH), 19.3 (CH_3), 16.3 (CH_2), 3.4 (CH_3) ppm; IR (ATR) ν: 2921, 2829, 1446, 1378, 1326, 1192, 1125, 1054, 958, 913, 809 cm^{-1}; MS (ESI) *m/z*: 391.5 $[2M+Na]^+$; HRMS (ESI) *m/z*: $[M+Na]^+$ calcd for $C_{11}H_{20}NaO_2^+$, 207.1356; found, 207.1355.

Aldehyde 26: A mixture of alkyne **25** (739 mg, 4.01 mmol), TsOH·H_2O (192 mg, 1.01 mmol), water (9 mL), isopropanol (1.6 mL), and THF (18 mL) was heated for 1 h under reflux. After letting the mixture cool to room temperature, brine was added, and the aqueous layer was extracted with ethyl acetate. The combined organic layers were dried over $MgSO_4$, and the solvents were removed under reduced pressure. The residue was purified by flash chromatography (isohexane/ethyl acetate, 20:1) to afford the unstable aldehyde **26** (498 mg, 90%).

26: R_f = 0.79 (isohexane/ethyl acetate, 3:1); $[\alpha]_D^{20}$ = –18.2 (*c* 0.80, $CHCl_3$); ^{1}H NMR (300 MHz, $CDCl_3$) δ 9.76 (t, *J* = 2.2 Hz, 1H), 2.50–2.36 (m, 1H), 2.30–2.11 (m, 4H), 1.77 (t, *J* = 2.5 Hz, 3H), 1.60–1.32 (m, 2H), 0.97 (d, *J* = 6.4 Hz, 3H) ppm; ^{13}C NMR (75 MHz, $CDCl_3$) δ 202.9 (CH), 78.6 (C), 76.1 (C), 50.8 (CH_2), 36.0 (CH_2), 27.4 (CH), 19.7 (CH_3), 16.5 (CH_2), 3.6 (CH_3) ppm; IR (ATR) ν: 2958, 2921, 2852, 2135, 2056, 1705, 1652, 1409, 1294, 1223, 1167, 1082, 927 cm^{-1}; MS (ESI) *m/z*: 139.1 $[M+H]^+$; HRMS (ESI) *m/z*: $[M+Na]^+$ calcd for $C_9H_{14}NaO^+$, 161.0937; found, 161.0936.

Keto aldehyde 27: In a pointed flask, a mixture of aldehyde **26** (810 mg, 5.86 mmol) and methyl vinyl ketone (**15**, 1.23 g, 17.6 mmol) cooled to 0 °C was treated successively with proline derivative **16** (156.6 mg, 0.586 mmol) and catechol **17** (213.5 mg, 1.172 mmol). After stirring for 89 h at 2 °C, the mixture was directly subjected to flash chromatography (pentane/diethyl ether, 4:1) to give the keto aldehyde **27** (1.16 g) as a rather unstable colorless oil that was immediately converted to dibromo olefin **28**. From another run, a sample of **27** was analyzed.

27: R_f = 0.15 (pentane/diethyl ether, 4:1); $[\alpha]_D^{25}$ = +32.9 (*c* 0.89, CH_2Cl_2); ^{1}H NMR (300 MHz, $CDCl_3$) δ 9.65 (d, *J* = 2.6 Hz, 1H), 2.60–2.47 (m, 1H), 2.45–2.32 (m, 1H), 2.25–2.16 (m, 2H), 2.16–2.12 (m, 4H), 2.11–2.01 (m, 1H), 1.99–1.85 (m, 1H), 1.81–1.75 (m, 4H), 1.69–1.53 (m, 1H), 1.47–1.35 (m, 1H), 0.99 (d, *J* = 6.8 Hz, 3H) ppm; ^{13}C NMR (75 MHz, $CDCl_3$) δ 208.0 (C), 205.1 (CH), 78.2 (C), 76.1 (C), 55.9 (CH), 41.4 (CH_2), 32.9 (CH_2), 32.6 (CH), 30.0 (CH_3), 19.7 (CH_2), 16.52 (CH_3), 16.49 (CH_2), 3.4 (CH_3) ppm; IR (ATR) ν: 2955, 2921, 2852, 2218, 2056, 1843, 1707, 1652, 1373, 1162, 943, 742 cm^{-1}; MS (ESI) *m/z*: 209.1 $[M+H]^+$.

Dibromo olefin 28: To a suspension of dibromomethyltriphenylphosphonium bromide (6.035 g, 11.72 mmol) in THF (50 mL) cooled to 0 °C was added sodium *tert*-butoxide (1.07 g, 11.1 mmol). After stirring for 10 min at 0 °C and 20 min at room temperature, the mixture was cooled to 0 °C and treated with a solution of keto aldehyde **27** (1.16 g, 5.56 mmol) in THF (12 mL). Stirring was continued for 1 h at 0 °C and 40 min at room temperature. Pentane (50 mL) was added, and the solids were removed by filtration through a plug of silica gel. The solvents were evaporated at reduced pressure, and purification of the residue by flash chromatography (isohexane/diethyl ether, 9:1) afforded dibromo olefin **28** (1.81 g, 85% over 2 steps, dr = 19:1 according to ^{1}H NMR integration) as a slightly yellow oil.

28: R_f = 0.20 (isohexane/diethyl ether, 15:1); $[\alpha]_D^{25}$ = +19.0 (*c* 0.90, CH_2Cl_2); ^{1}H NMR (600 MHz, $CDCl_3$) δ 6.16 (d, *J* = 10.5 Hz, 1H), 2.39–2.49 (m, 2H), 2.35 (tt, *J* = 10.5, 4.4 Hz, 1H), 2.24–2.17 (m, 1H), 2.16 (s, 3H), 2.17–2.09 (m, 1H), 1.78 (t, *J* = 2.5 Hz, 3H), 1.80–1.70 (m, 2H), 1.62–1.52 (m, 2H), 1.34–1.27 (m, 1H), 0.88 (d, *J* = 6.6 Hz, 3H) ppm; ^{13}C NMR (150 MHz, $CDCl_3$) δ 208.3 (C), 140.1 (CH), 89.4 (C), 78.7 (C), 75.8 (C), 47.3 (CH), 41.2 (CH_2), 35.8 (CH), 33.9 (CH_2), 30.2 (CH_3), 25.8 (CH_2), 16.6 (CH_2), 15.1 (CH_3), 3.5 (CH_3) ppm; IR (ATR) ν: 2958, 2919, 2111, 1990, 1917, 1843, 1770, 1714, 1684, 1652, 1618, 1435, 1362, 1257, 1162, 979, 800, 761 cm^{-1}; MS (ESI) *m/z*: 380.0 $[M+NH_4]^+$.

Enediyne 7: To a suspension of methyltriphenylphosphonium bromide (391 mg, 1.09 mmol) in THF (10 mL) was added a solution of BuLi in hexane (1.6 M, 0.6 mL, 0.96 mmol) over 30 min at room temperature. The mixture was stirred at room temperature for 30 min, a solution of dibromo olefin **28** (173 mg, 0.475 mmol) in THF (1.5 mL) was added, and the resulting mixture was heated to reflux for 1 h. After cooling to −78 °C, a solution of BuLi in

hexane (1.6 M, 0.65 mL, 1.04 mmol) was added, and stirring was continued at −78 °C for 90 min. The mixture was filtered through a plug of silica gel with pentane (200 mL), and the solvent was removed under reduced pressure. Purification of the residue by flash chromatography (pentane) gave enediyne **7** (86.0 mg, 90%) as a colorless oil.

7: R_f = 0.21 (pentane); $[\alpha]_D^{20}$ = +8.3 (*c* 0.89, CH_2Cl_2); ^{1}H NMR (500 MHz, $CDCl_3$) δ 4.75–4.69 (m, 2H), 2.42–2.35 (m, 1H), 2.29–2.21 (m, 1H), 2.21–2.14 (m, 2H), 2.13–2.06 (m, 1H), 2.04 (d, *J* = 2.5 Hz, 1H), 1.78 (t, *J* = 2.5 Hz, 3H), 1.74 (s, 3H), 1.73–1.61 (m, 3H), 1.59–1.53 (m, 1H), 1.53–1.43 (m, 1H), 0.94 (d, *J* = 6.6 Hz, 3H) ppm; ^{13}C NMR (125 MHz, $CDCl_3$) δ 145.4 (C), 110.2 (CH_2), 85.3 (C), 78.8 (C), 75.6 (C), 70.5 (CH), 35.8 (CH_2), 35.7 (CH), 34.8 (CH), 34.4 (CH_2), 30.9 (CH_2), 22.5 (CH_3), 16.4 (CH_2), 14.8 (CH_3), 3.5 (CH_3) ppm; IR (ATR) ν: 3305, 3074, 2962, 2922, 2858, 2109, 1648, 1447, 1377, 1326, 1258, 888, 631 cm^{-1}; GC–MS: *m/z* = 187 $[M–Me]^+$.

Dienediyne 8: To a suspension of 5-hexenyltriphenylphosphonium bromide (1.80 g, 4.23 mmol) in THF (40 mL) was added a solution of BuLi in hexane (1.6 M, 2.6 mL, 4.2 mmol) over 30 min at room temperature. The mixture was stirred at room temperature for 30 min, a solution of dibromo olefin **28** (734 mg, 2.02 mmol) in THF (6 mL) was added, and the resulting mixture was heated to reflux for 3 h. After cooling to −78 °C, a solution of BuLi in hexane (1.6 M, 2.8 mL, 4.5 mmol) was added, and stirring was continued −78 °C for 2 h. The mixture was filtered through a plug of silica gel with pentane (400 mL), and the solvents were removed under reduced pressure. Purification of the residue by flash chromatography (pentane) gave dienediyne **8** (353 mg, 65%, 1.3:1 mixture of *E* and *Z* olefin isomers) as a colorless oil.

8: R_f = 0.23 (pentane); $[\alpha]_D^{20}$ = +8.9 (*c* 2.09, CH_2Cl_2); ^{1}H NMR (300 MHz, $CDCl_3$) δ 5.90–5.73 (m, 1H), 5.23–5.09 (m, 1H), 5.05–4.91 (m, 2H), 2.39–2.29 (m, 1H), 2.22–2.11 (m, 3H), 2.11–1.96 (m, 6H), 1.77 (t, *J* = 2.5 Hz, 3H), 1.72–1.39 (m, 10H), 0.94 (d, *J* = 6.4 Hz, 3H) ppm; IR (ATR) ν: 3304, 3075, 2958, 2922, 2856, 2109, 2029, 1640, 1444, 1378, 991, 909, 626 cm^{-1}; GC–MS: *m/z* = 255 $[M–Me]^+$.

(−)-Isoguaiene (1) from 8: : In a round-bottom 100 mL flask equipped with a Vigreux column, which was connected via a two-neck (Anschütz) attachment to a reflux condenser, a solution of catalyst **29** (98.9 mg, 0.120 mmol) in dichloromethane (30 mL) was heated to reflux (oil bath temperature 55 °C). A solution of dienediyne **8** (325.0 mg, 1.2 mmol) in dichloromethane (30 mL) was added through one neck of the Anschütz attachment over 3 h, and the resulting mixture was stirred for 5 h in total at reflux. Further portions of a solution of catalyst **29** (49.5 mg, 0.0602 mmol) in dichloromethane (5 mL) were added after 1, 2, and 3 h of reflux. The mixture was allowed to cool to room temperature, the solvent was carefully removed under reduced pressure, and then the residue (**6**, 149 mg, 0.736 mmol) was taken up in pentane and filtered through a pad of silica gel. The solvent was carefully removed under reduced pressure, the residue was taken up in a mixture of benzene and ethanol (1:1, 10 mL), and $(PPh_3)_3RhCl$ (102.1 mg, 0.110 mmol) was added. After stirring for 4.5 h under hydrogen atmosphere (balloon), pentane (60 mL) was added, and the mixture was washed with water (2 × 15 mL) and dried over $MgSO_4$. The solvents were carefully removed under reduced pressure, and the residue was purified by flash chromatography (pentane) to give (−)-isoguaiene **1** (103.3 mg, 42% over 2 steps) as a colorless oil.

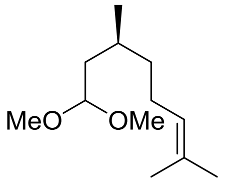
MeO
OMe

10

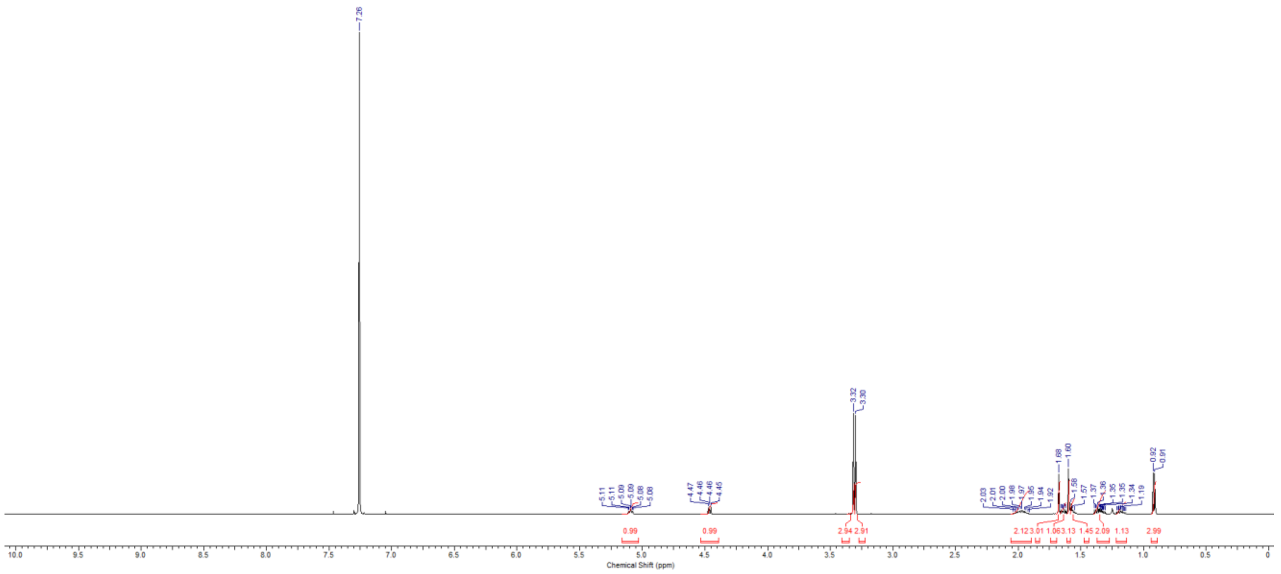
7.26
3.32
3.30
0.92
0.91
0.99
0.99
2.94
2.91
2.12
3.01
1.06
3.13
1.45
2.09
1.13
2.99
Chemical Shift (ppm)

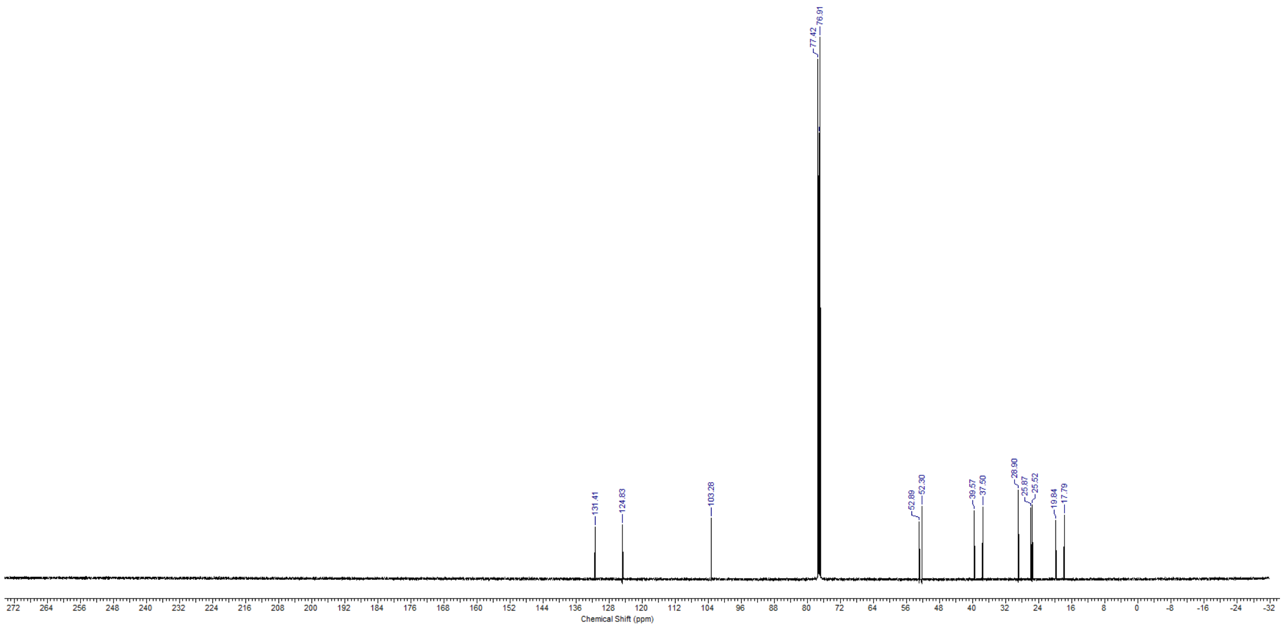
77.42
76.91
131.41
124.83
103.28
52.89
52.30
39.57
37.50
28.90
25.87
25.52
19.84
17.79
Chemical Shift (ppm)

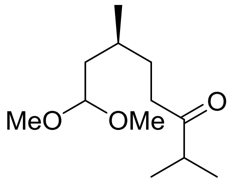
MeO
OMe
O

12

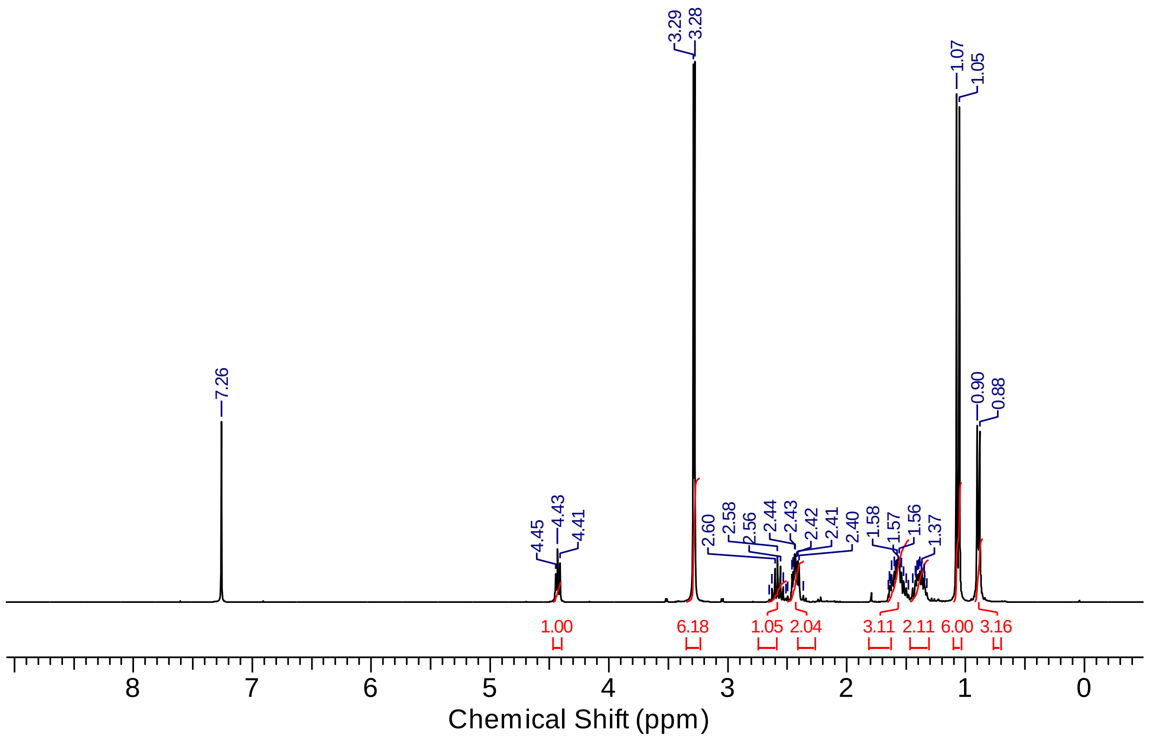
7.26
4.45
4.43
4.41
3.29
3.28
2.60
2.58
2.56
2.44
2.43
2.42
2.41
2.40
1.58
1.57
1.56
1.37
1.07
1.05
0.90
0.88
1.00
6.18
1.05
2.04
3.11
2.11
6.00
3.16
8
7
6
5
4
3
2
1
0
Chemical Shift (ppm)

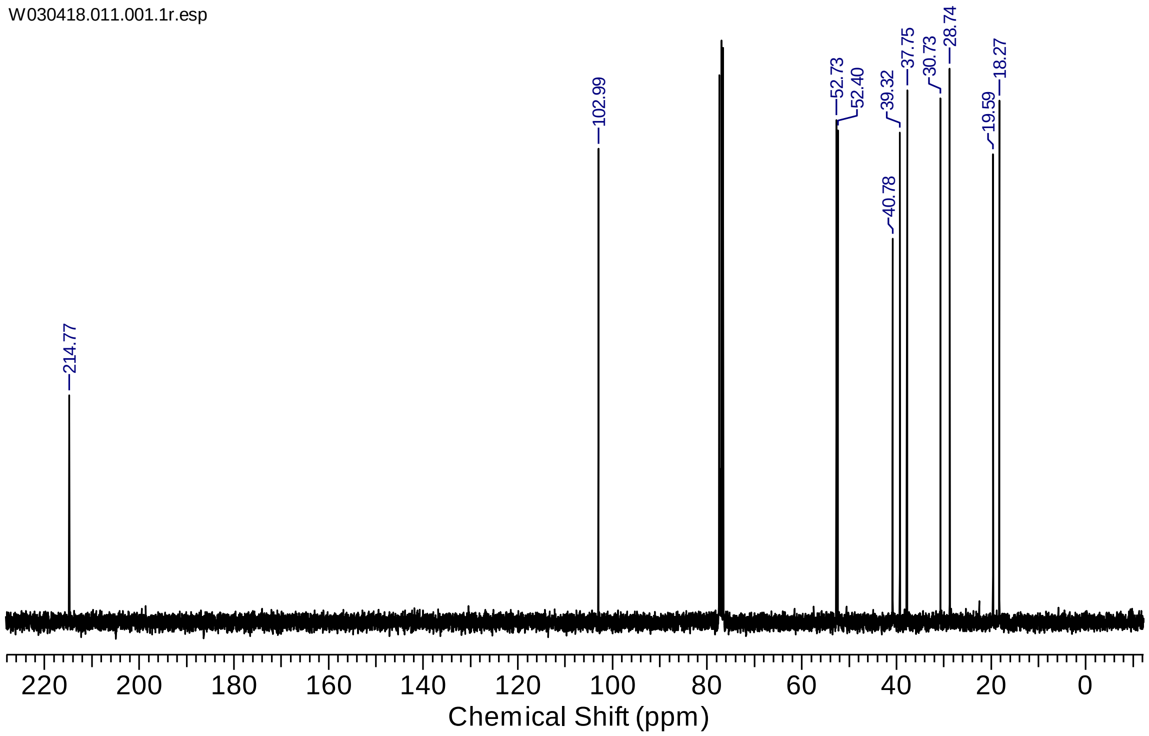
W030418.011.001.1r.esp
214.77
102.99
52.73
52.40
40.78
39.32
37.75
30.73
28.74
19.59
18.27
220
200
180
160
140
120
100
80
60
40
20
0
Chemical Shift (ppm)

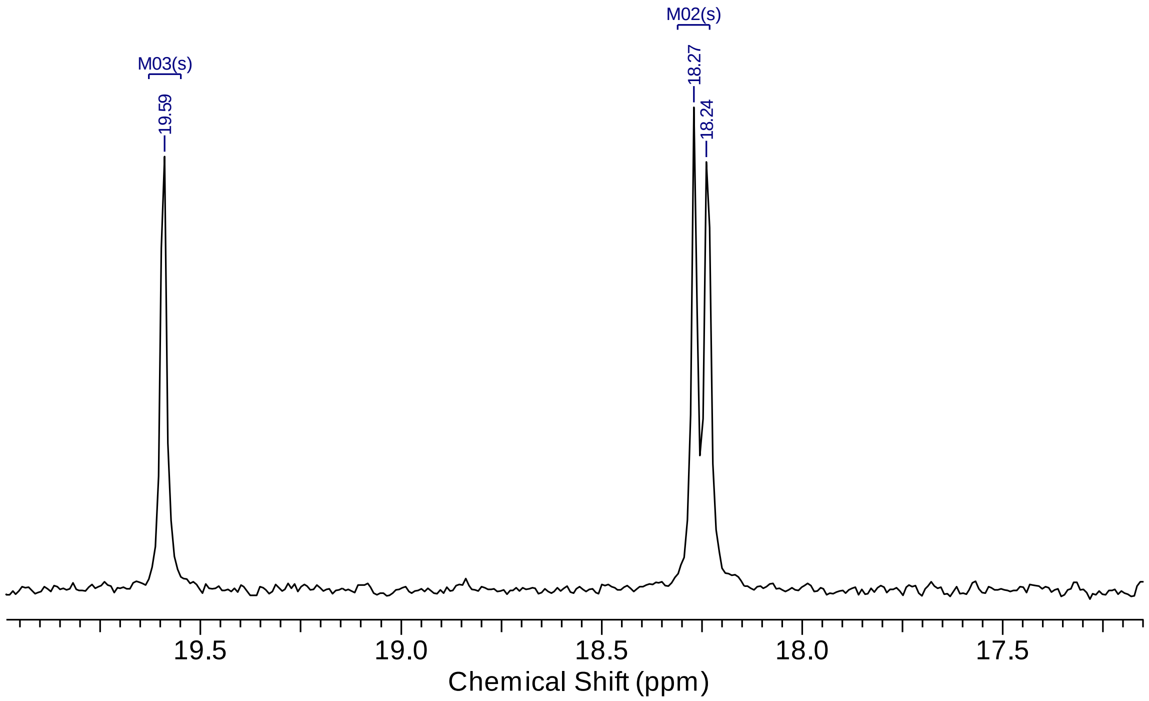
M03(s)
19.59
M02(s)
18.27
18.24
19.5
19.0
18.5
18.0
17.5
Chemical Shift (ppm)

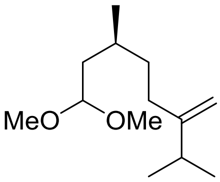
MeO
OMe

14

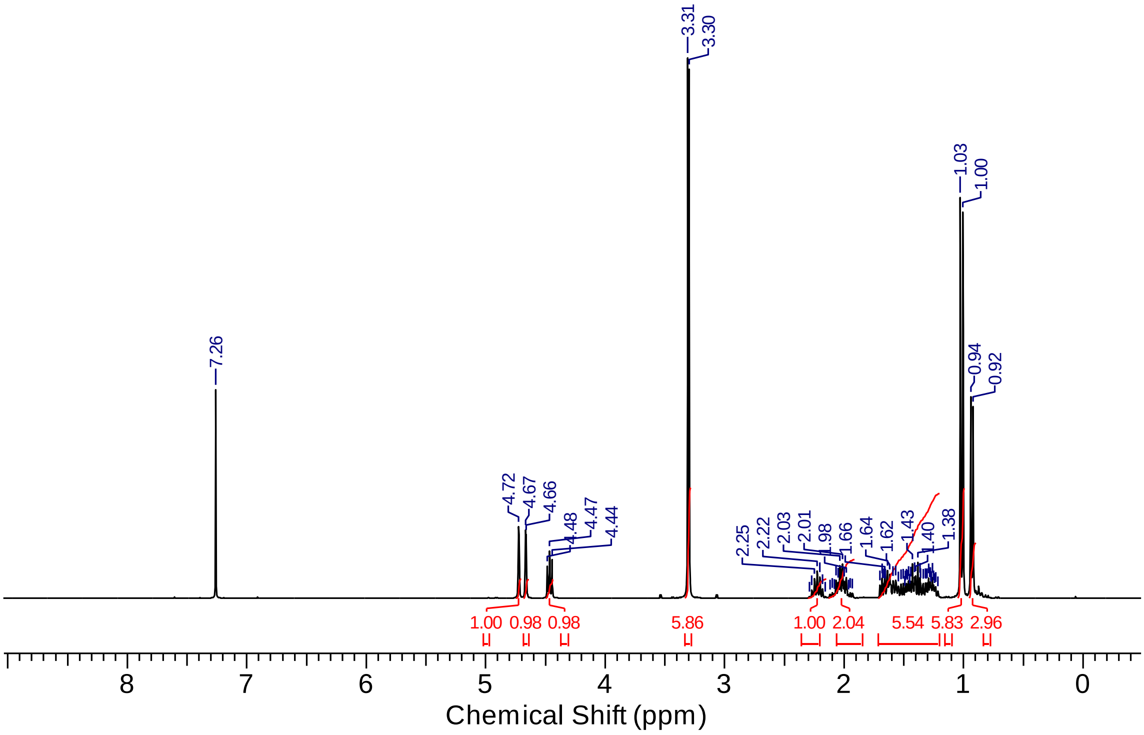
7.26
4.72
4.67
4.66
4.48
4.47
4.44
3.31
3.30
2.25
2.22
2.03
2.01
1.98
1.66
1.64
1.62
1.43
1.40
1.38
1.03
1.00
0.94
0.92
1.00 0.98 0.98 5.86 1.00 2.04 5.54 5.83 2.96
8 7 6 5 4 3 2 1 0
Chemical Shift (ppm)

W090418.011.001.1r.esp

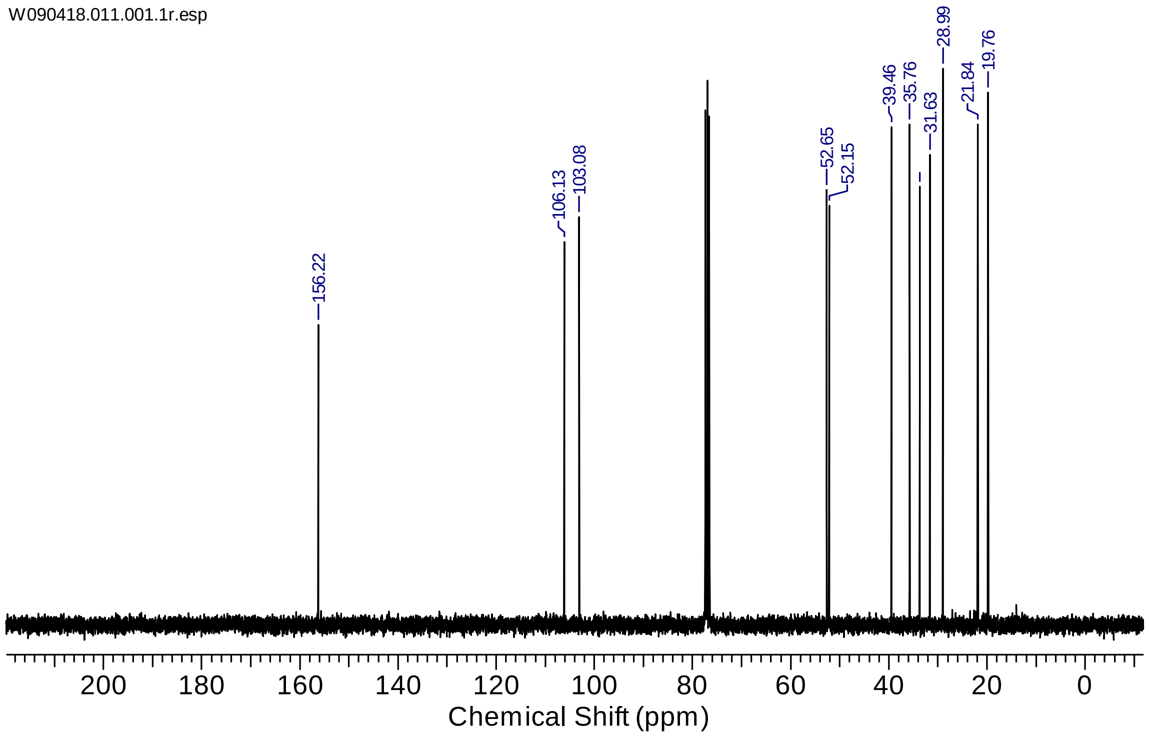

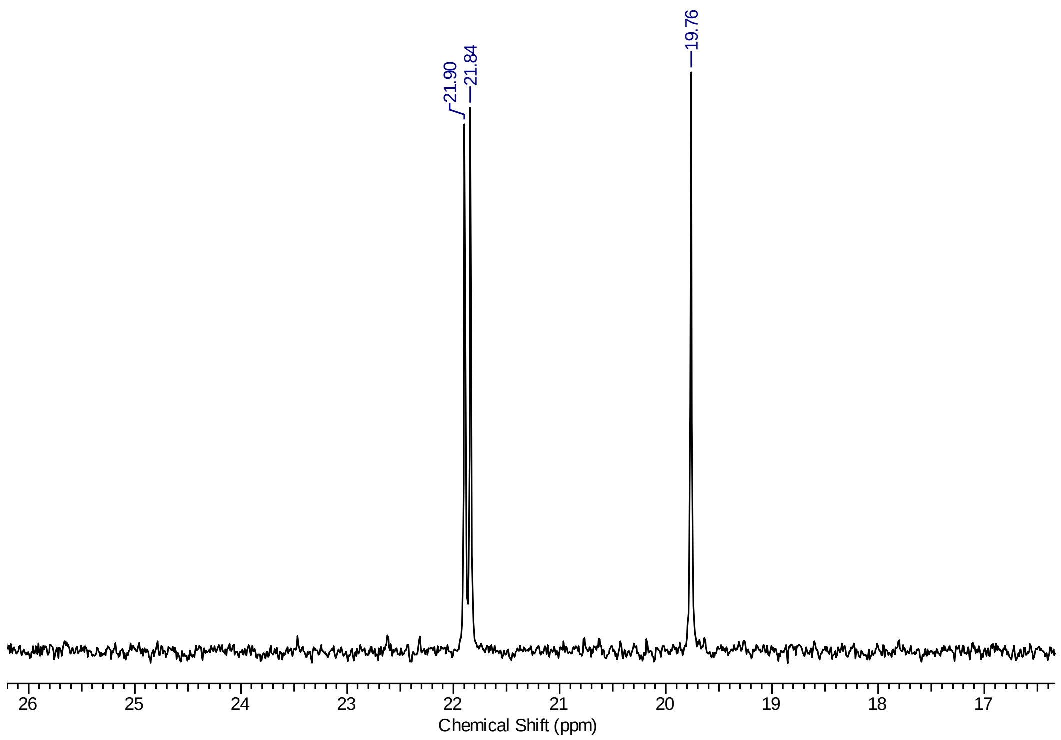

21.90
21.84
19.76
26
25
24
23
22
21
20
19
18
17
Chemical Shift (ppm)

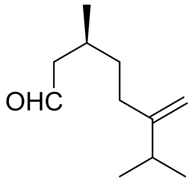
OHC

4

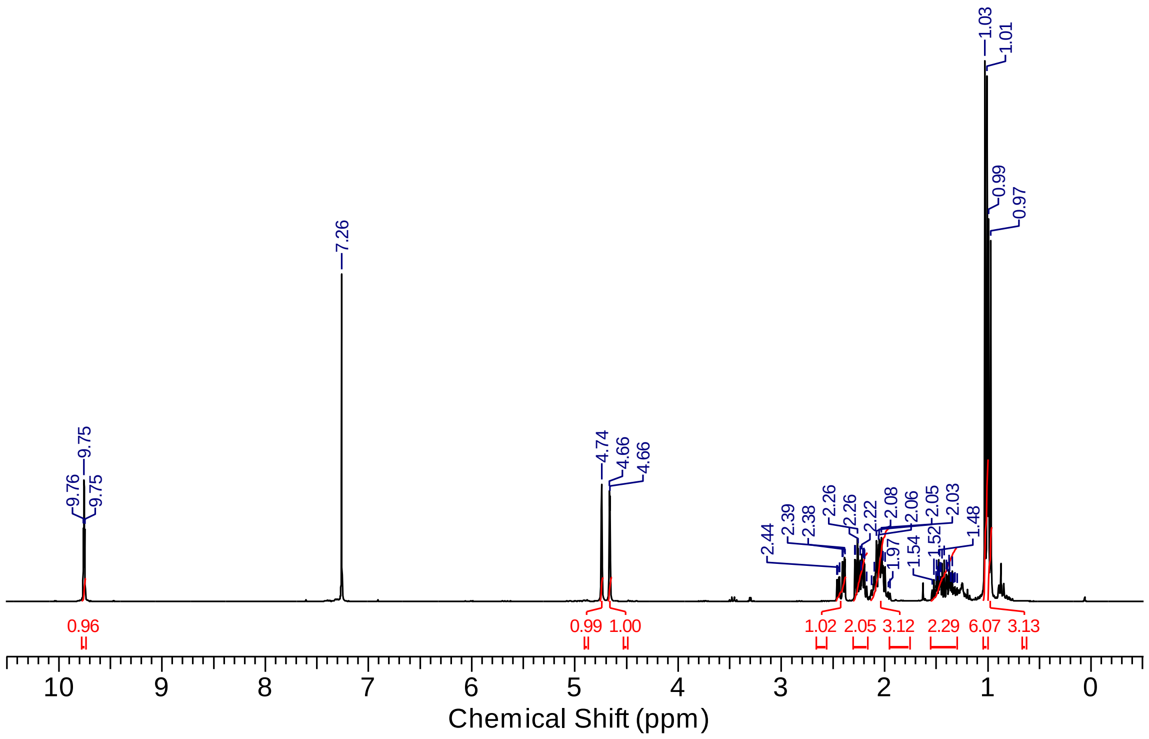
9.76
9.75
9.75
7.26
4.74
4.66
4.66
2.44
2.39
2.38
2.26
2.26
2.22
2.08
2.06
2.05
2.03
1.97
1.54
1.52
1.48
1.03
1.01
0.99
0.97
0.96
0.99
1.00
1.02
2.05
3.12
2.29
6.07
3.13
10
9
8
7
6
5
4
3
2
1
0
Chemical Shift (ppm)

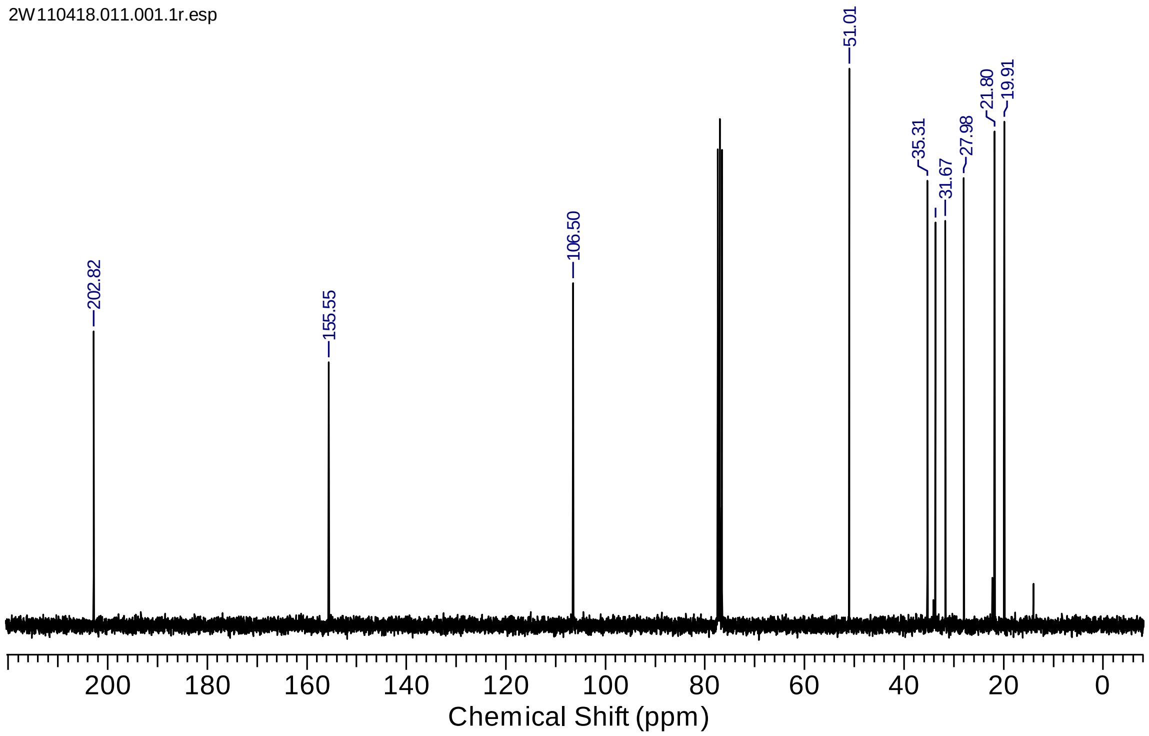
2W110418.011.001.1r.esp
202.82
155.55
106.50
51.01
35.31
31.67
27.98
21.80
19.91
200
180
160
140
120
100
80
60
40
20
0
Chemical Shift (ppm)

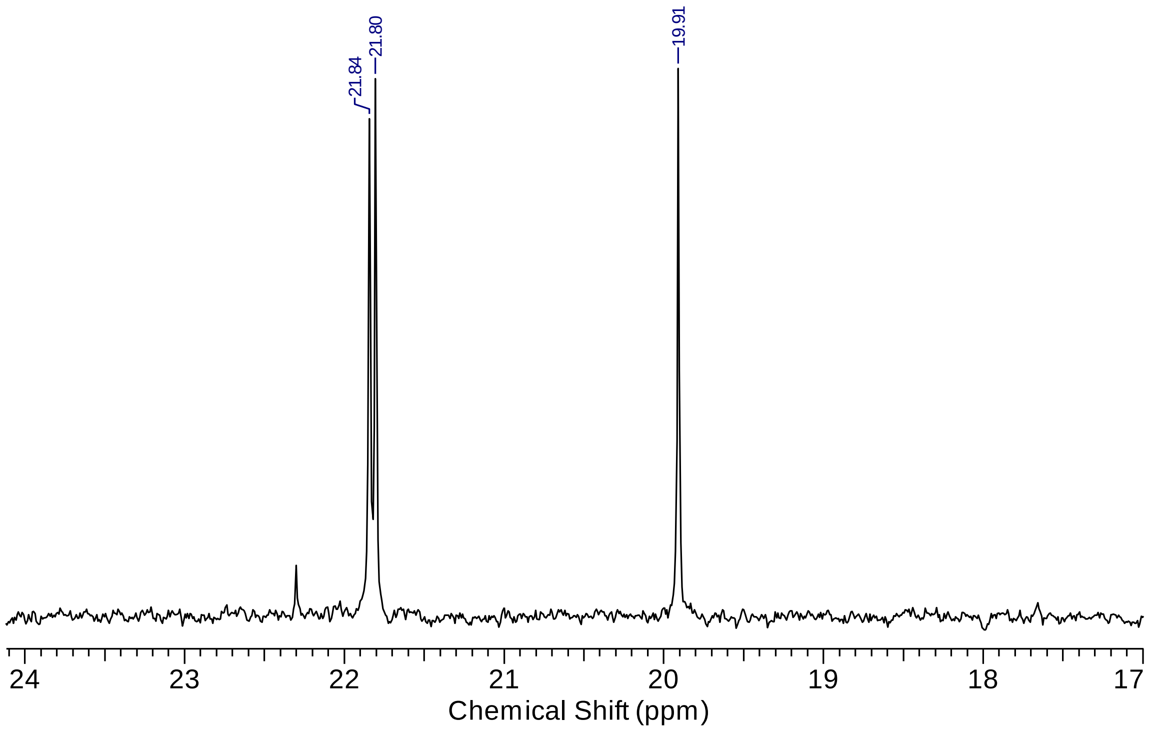
21.84
21.80
19.91
24
23
22
21
20
19
18
17
Chemical Shift (ppm)

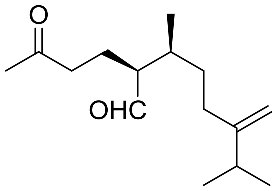
O
OHC

18

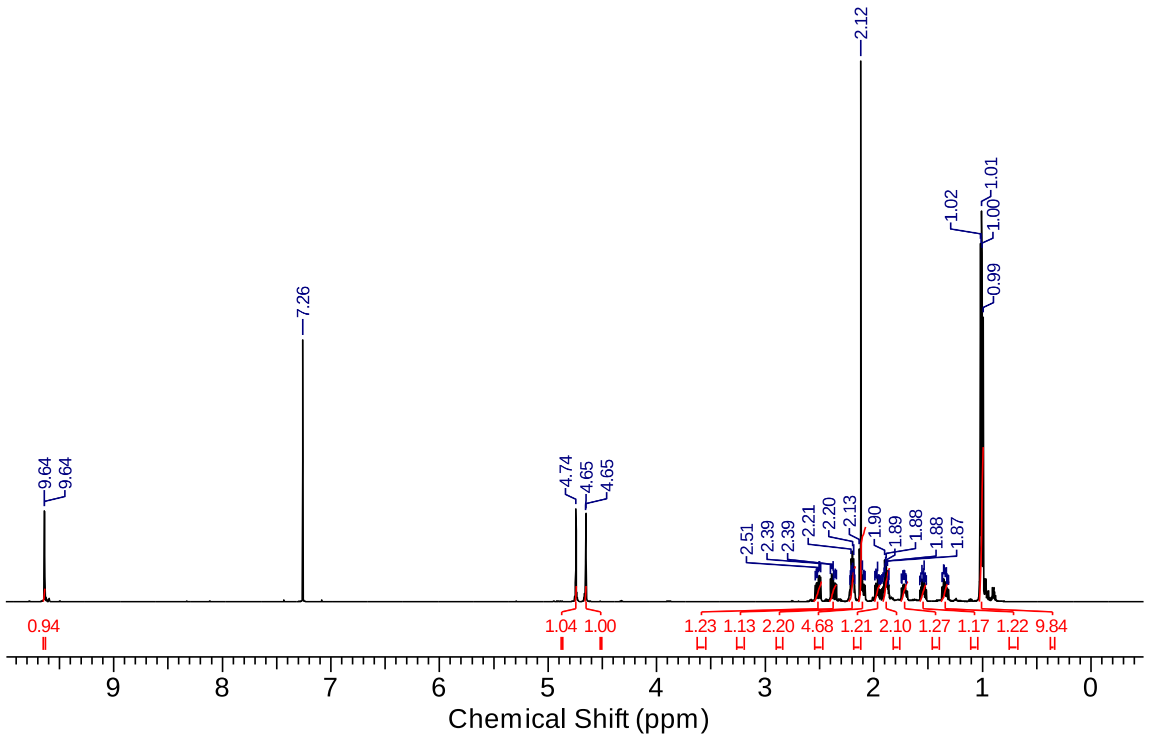
9.64
9.64
7.26
4.74
4.65
4.65
2.12
2.51
2.39
2.39
2.21
2.20
2.13
1.90
1.89
1.88
1.88
1.87
1.02
1.01
1.00
0.99
0.94
1.04 1.00
1.23 1.13 2.20 4.68 1.21 2.10 1.27 1.17 1.22 9.84
9
8
7
6
5
4
3
2
1
0
Chemical Shift (ppm)

W140418.010.001.1r.esp

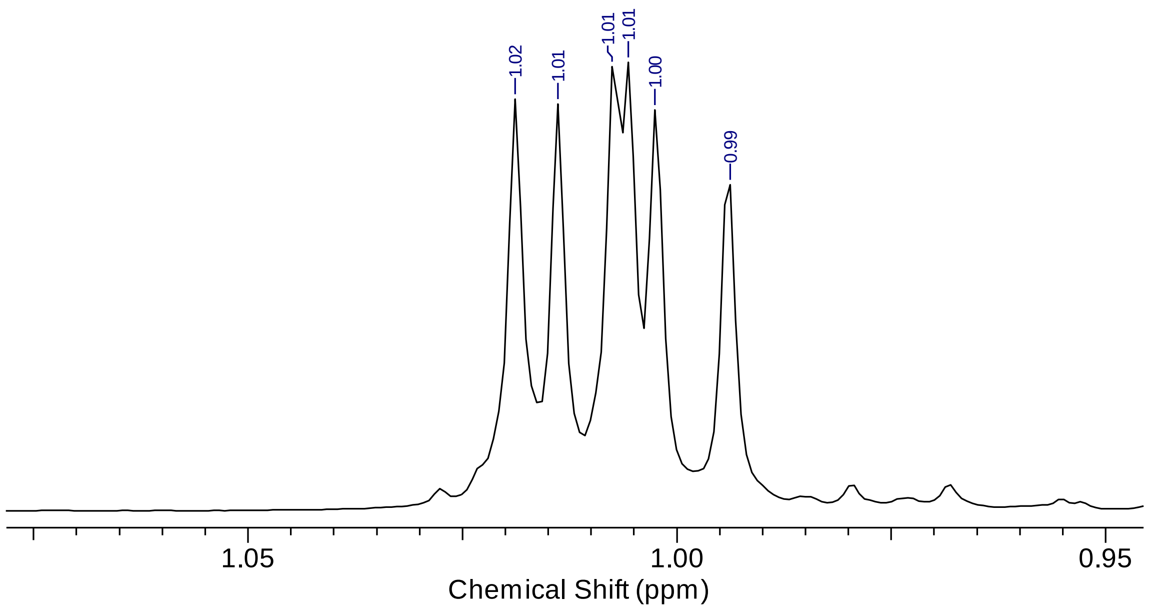

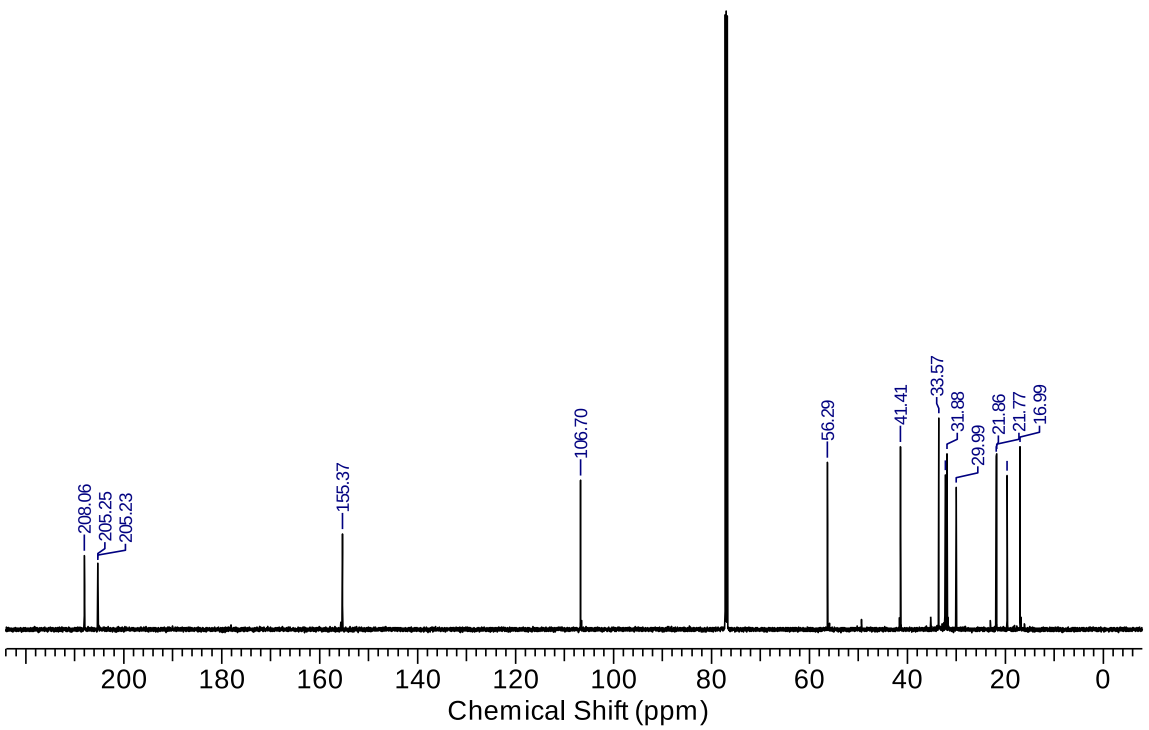
208.06
205.25
205.23
155.37
106.70
56.29
41.41
33.57
31.88
29.99
21.86
21.77
16.99
200
180
160
140
120
100
80
60
40
20
0
Chemical Shift (ppm)

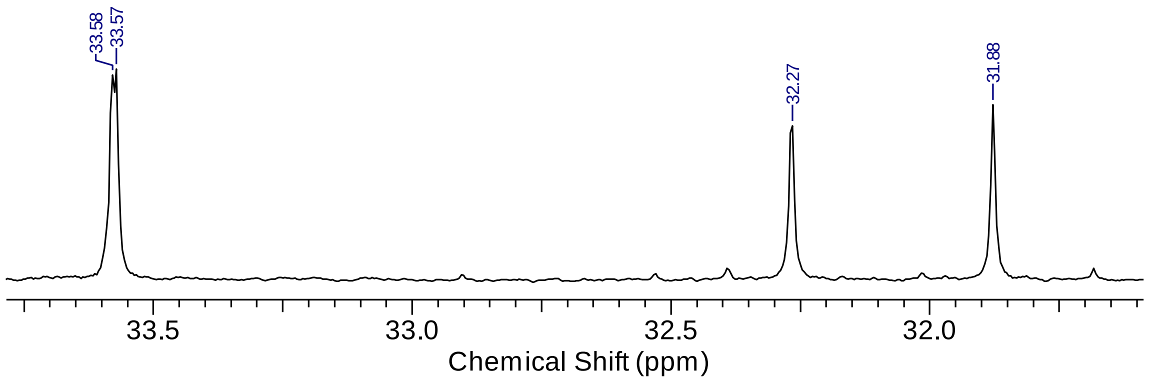
33.58
33.57
32.27
31.88
33.5
33.0
32.5
32.0
Chemical Shift (ppm)

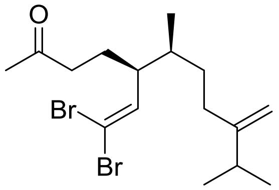
O
Br
Br

20

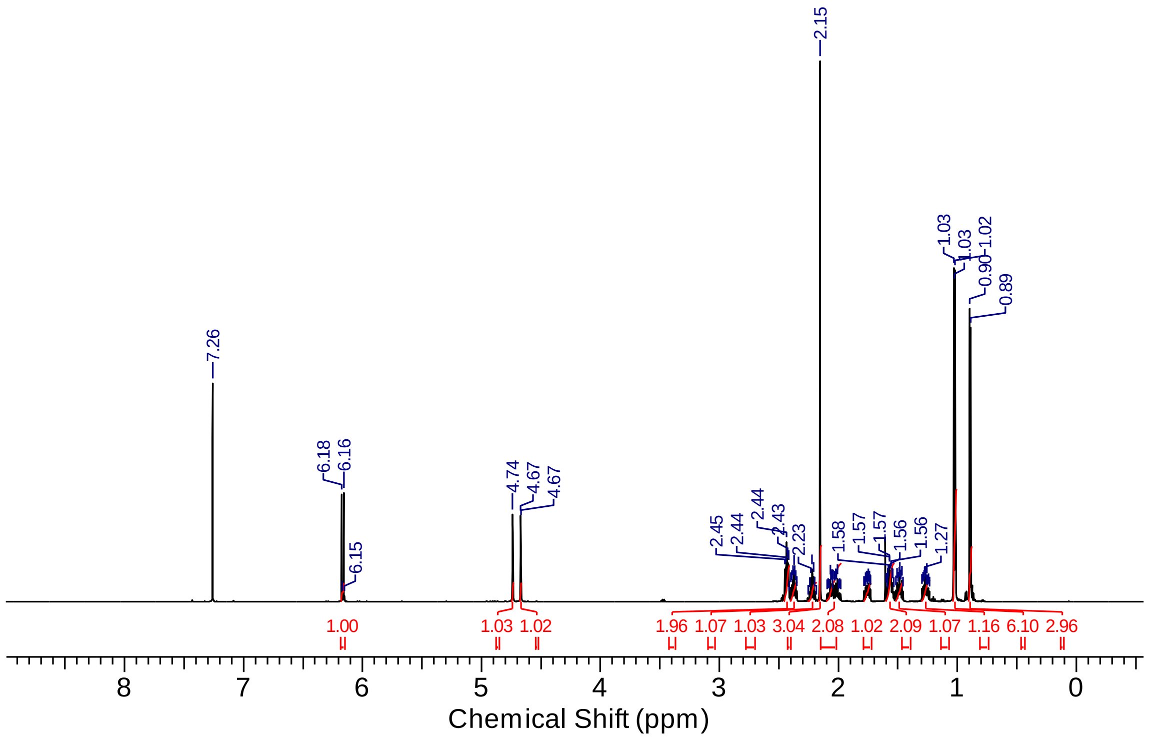
7.26
6.18
6.16
6.15
4.74
4.67
4.67
2.45
2.44
2.44
2.43
2.23
2.15
1.58
1.57
1.57
1.56
1.56
1.27
1.03
1.03
1.02
0.90
0.89
1.00
1.03 1.02
1.96 1.07 1.03 3.04 2.08 1.02 2.09 1.07 1.16 6.10 2.96
8 7 6 5 4 3 2 1 0
Chemical Shift (ppm)

W190418.010.002.1r.esp

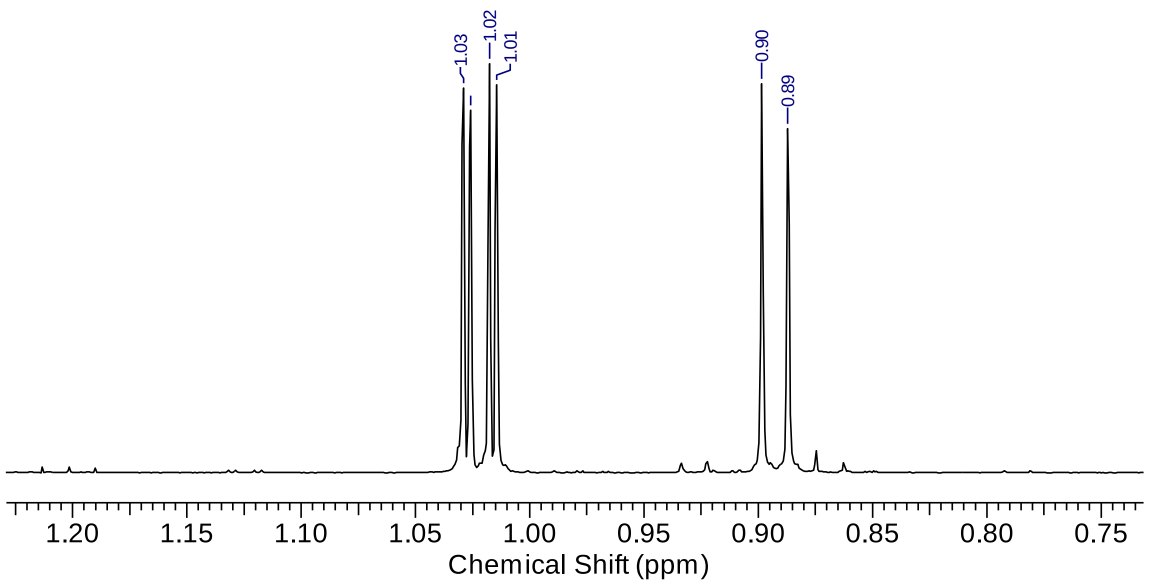

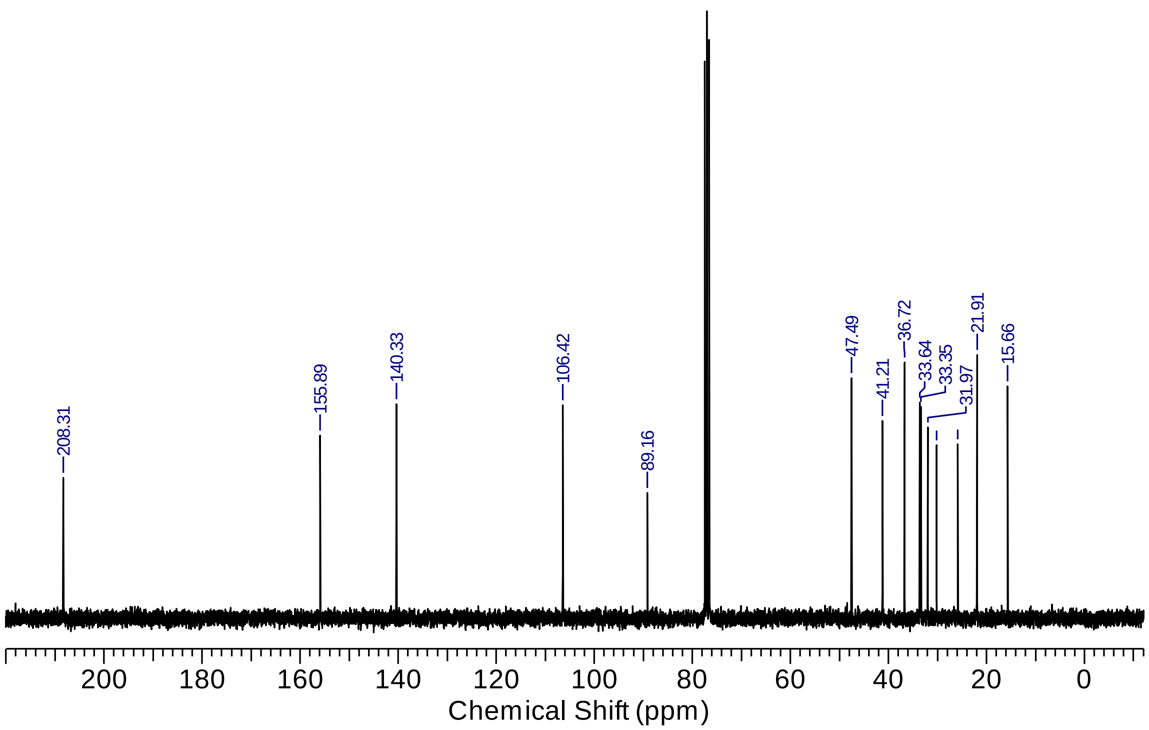
208.31
155.89
140.33
106.42
89.16
47.49
41.21
36.72
33.64
33.35
31.97
21.91
15.66
200
180
160
140
120
100
80
60
40
20
0
Chemical Shift (ppm)

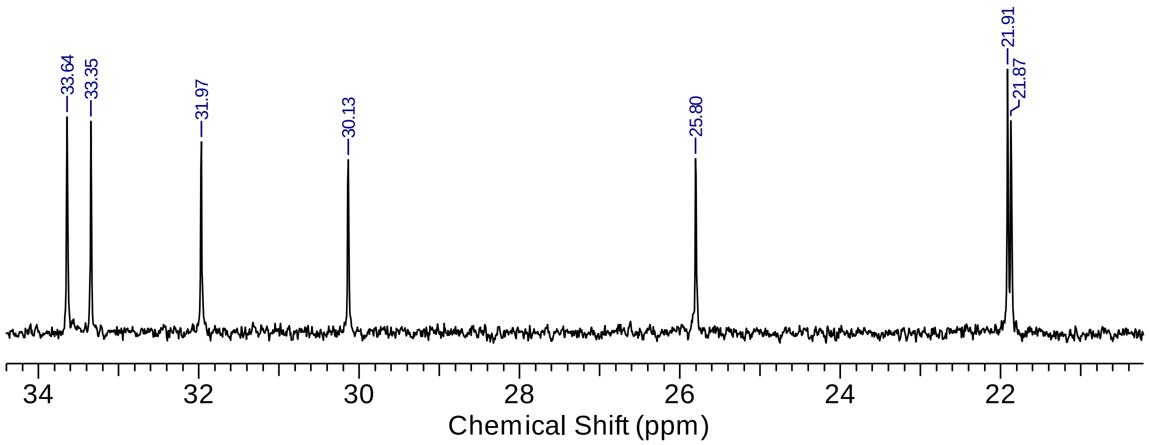
33.64
33.35
31.97
30.13
25.80
21.91
21.87
34
32
30
28
26
24
22
Chemical Shift (ppm)

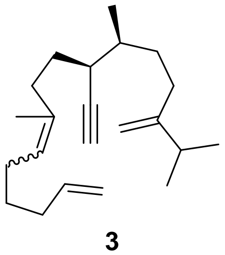

3

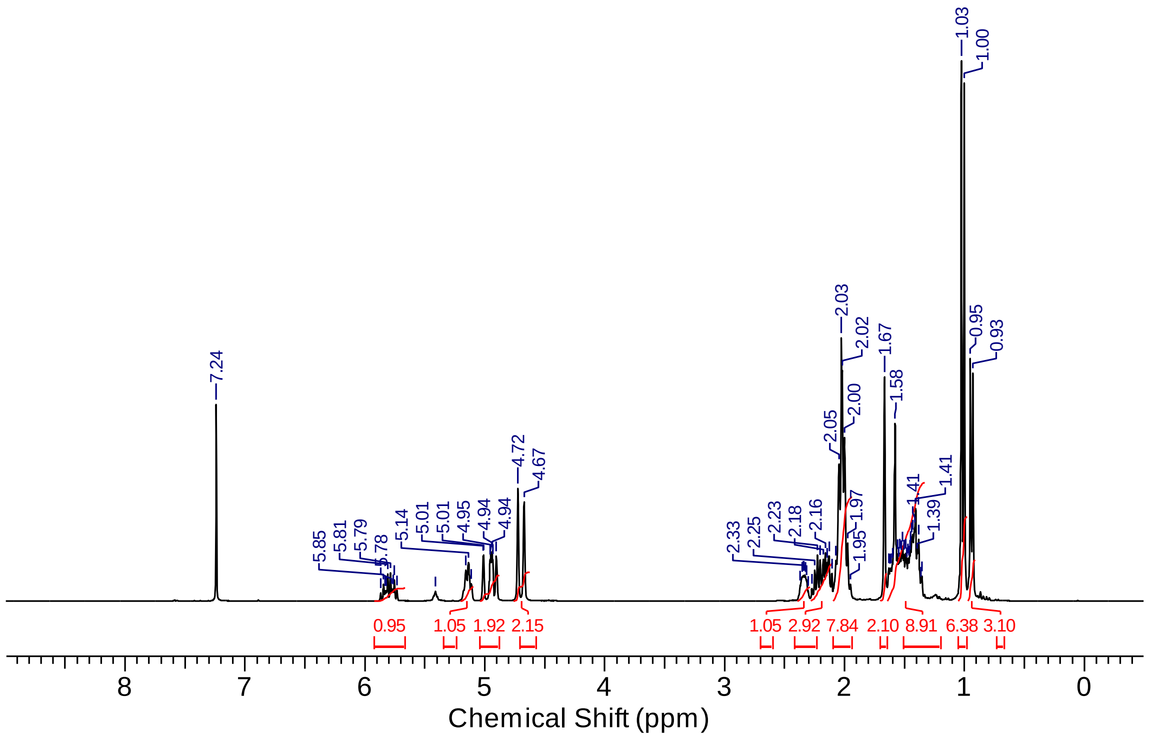

7.24
5.85
5.81
5.79
5.78
5.14
5.01
5.01
4.95
4.94
4.94
4.72
4.67
2.33
2.25
2.23
2.18
2.16
2.05
2.03
2.02
2.00
1.97
1.95
1.67
1.58
1.41
1.41
1.39
1.03
1.00
0.95
0.93
0.95
1.05
1.92
2.15
1.05
2.92
7.84
2.10
8.91
6.38
3.10
8
7
6
5
4
3
2
1
0
Chemical Shift (ppm)

W240418.011.001.1r.esp

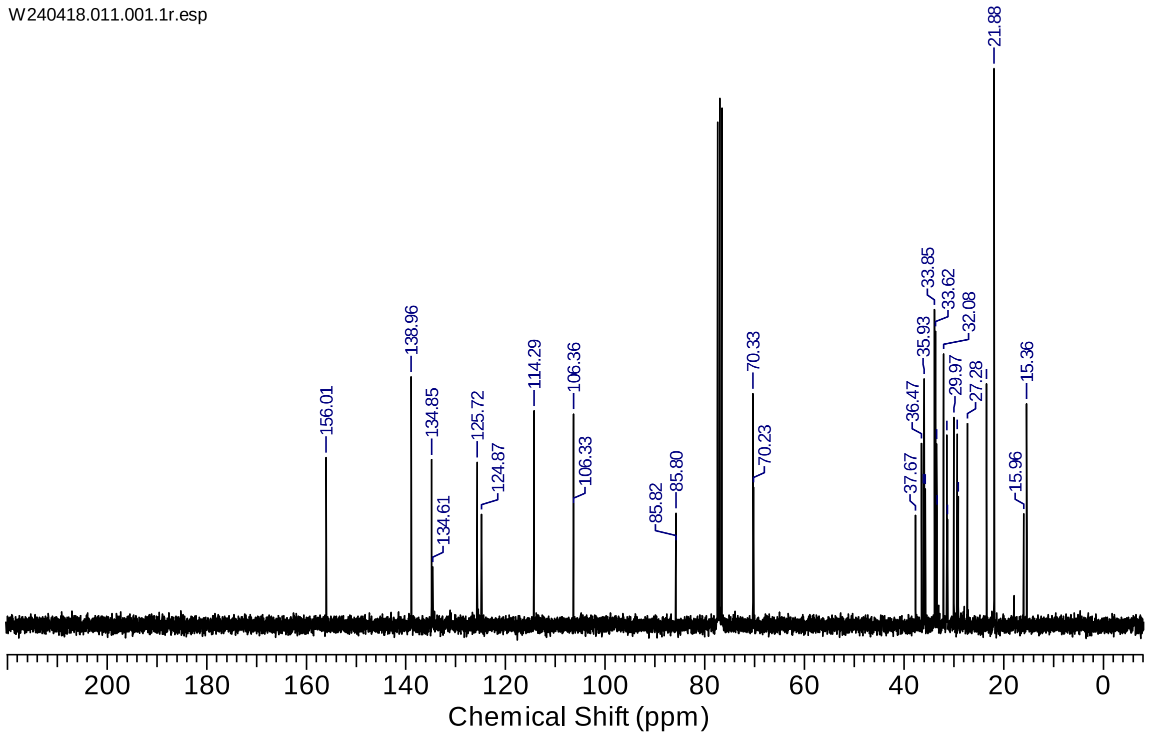

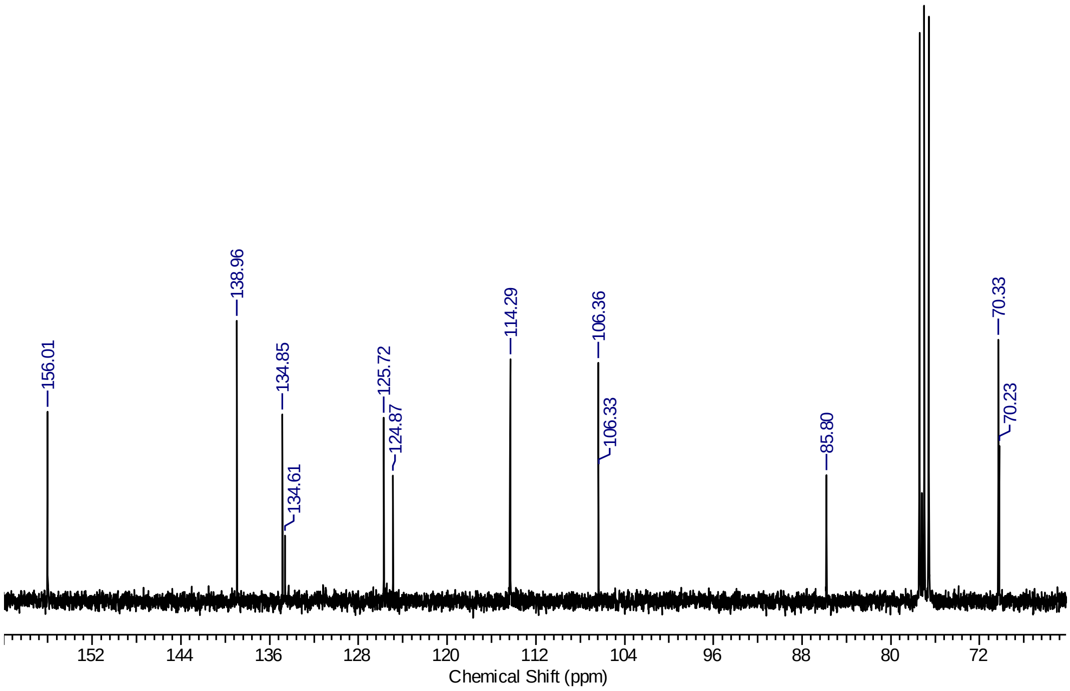
156.01
138.96
134.85
134.61
125.72
124.87
114.29
106.36
106.33
85.80
70.33
70.23
152
144
136
128
120
112
104
96
88
80
72
Chemical Shift (ppm)

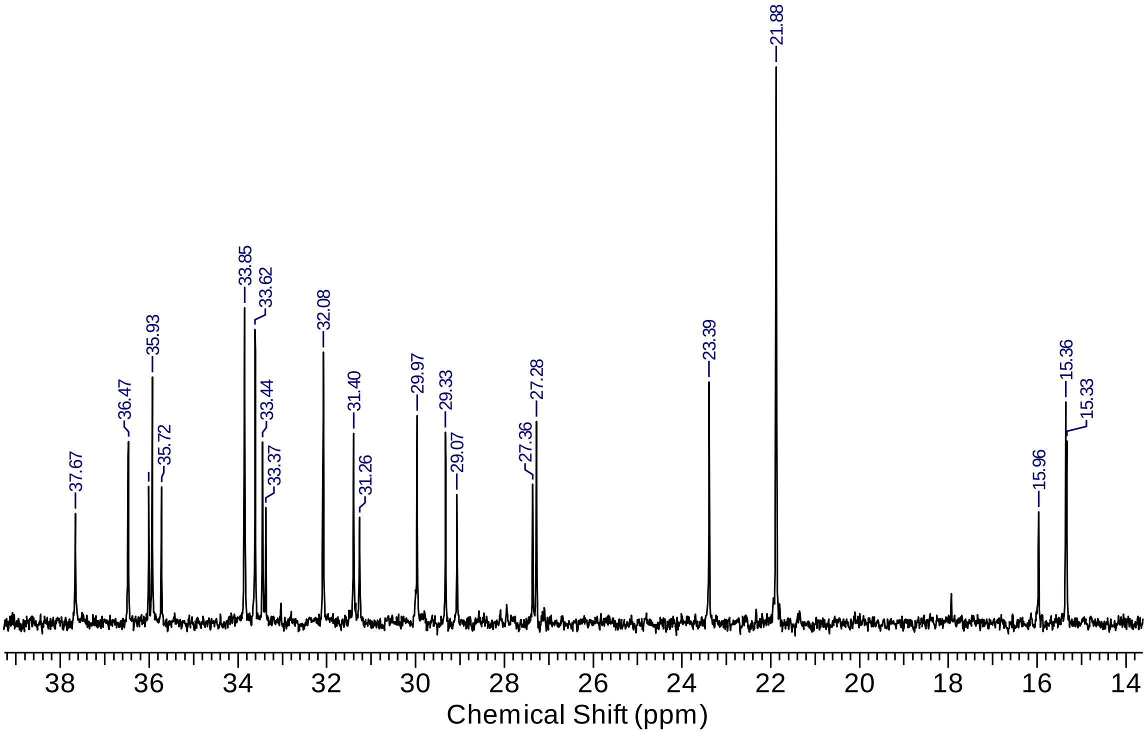
37.67
36.47
35.93
35.72
33.85
33.62
33.44
33.37
32.08
31.40
31.26
29.97
29.33
29.07
27.36
27.28
23.39
21.88
15.96
15.36
15.33
38
36
34
32
30
28
26
24
22
20
18
16
14
Chemical Shift (ppm)

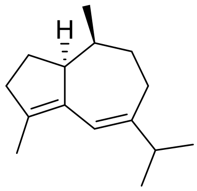

1

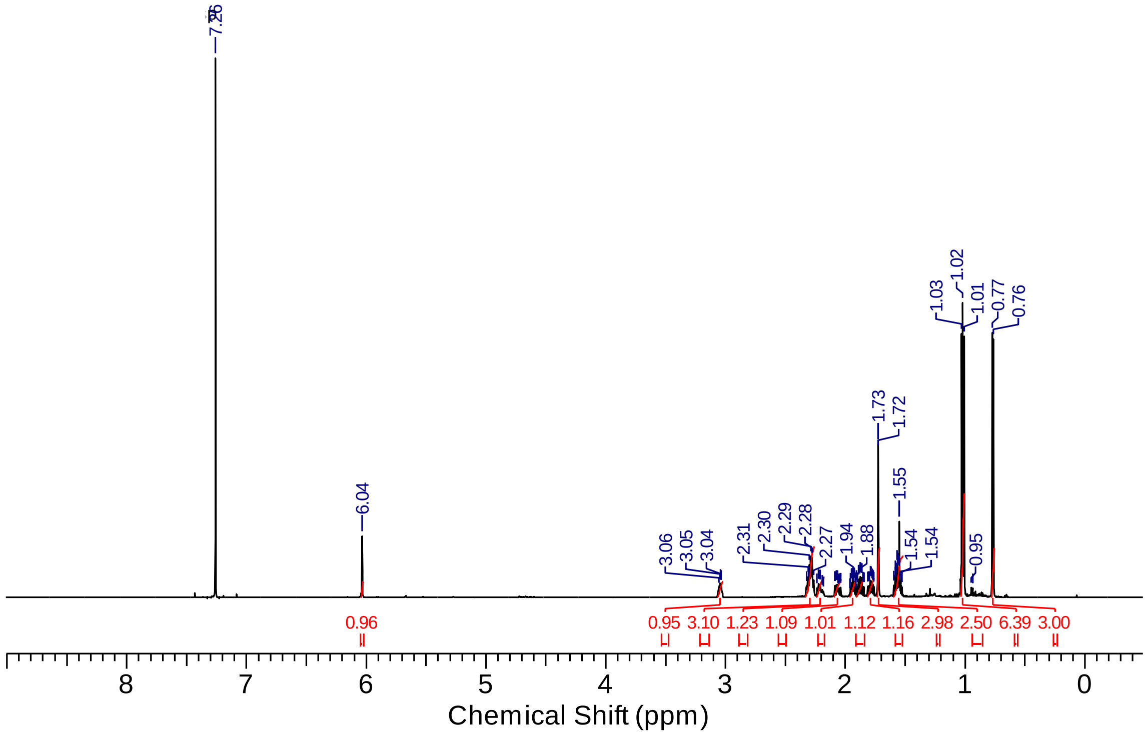

7.26
6.04
3.06
3.05
3.04
2.31
2.30
2.29
2.28
2.27
1.94
1.88
1.73
1.72
1.55
1.54
1.54
1.03
1.02
1.01
0.95
0.77
0.76
0.96
0.95 3.10 1.23 1.09 1.01 1.12 1.16 2.98 2.50 6.39 3.00
8 7 6 5 4 3 2 1 0
Chemical Shift (ppm)

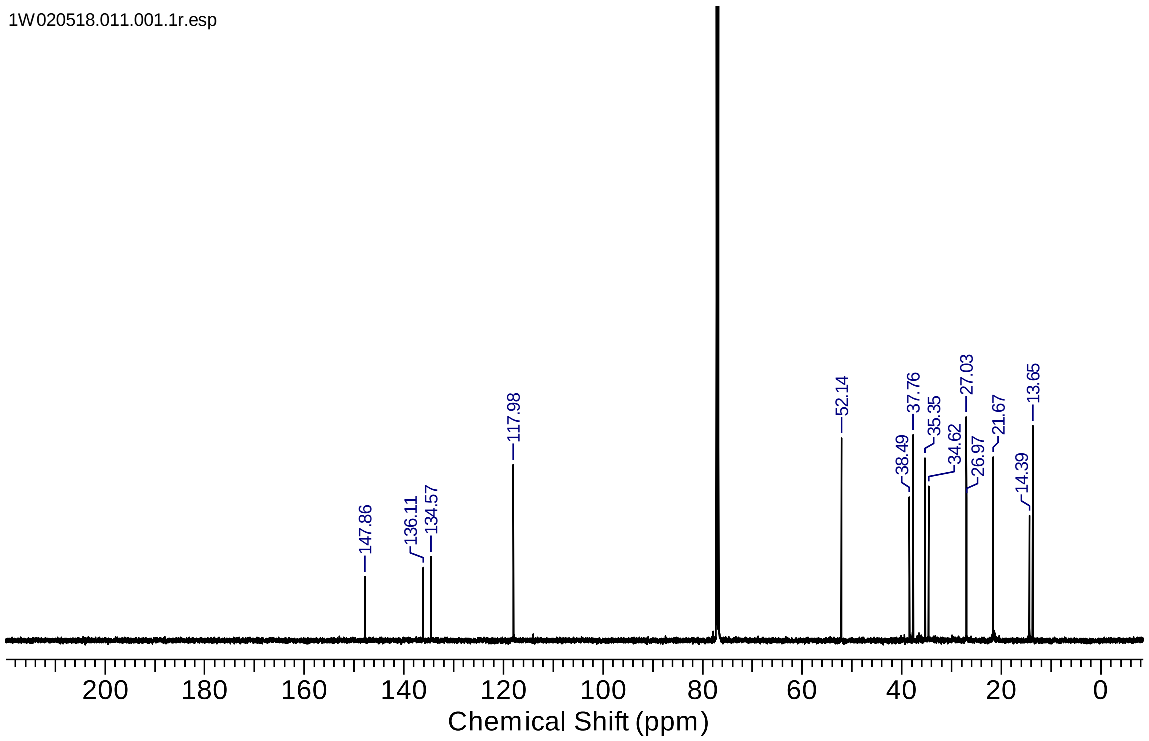
1W020518.011.001.1r.esp
147.86
136.11
134.57
117.98
52.14
38.49
37.76
35.35
34.62
27.03
26.97
21.67
14.39
13.65
200 180 160 140 120 100 80 60 40 20 0
Chemical Shift (ppm)

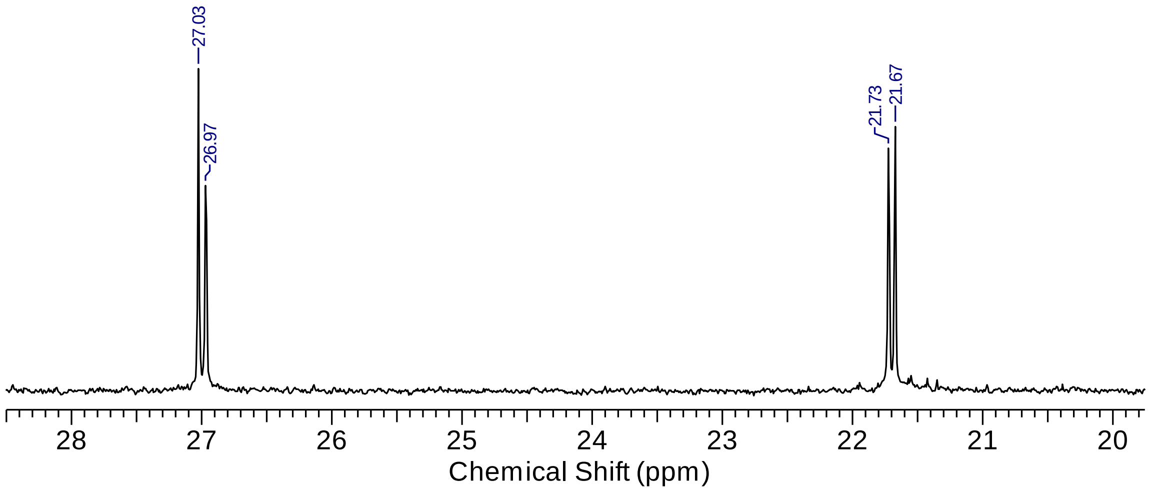

Table S1. Comparison of ^{13}C NMR Data [ppm] for **1** with Literature[a] Data.

Literature[b]	This Work[c]	Literature[b]	This Work[c]	Literature[b]	This Work[c]
147.8 (C)	147.9 (C)	38.5 (CH)	38.5 (CH)	26.9 (CH_2)	26.97 (CH_2)
136.1 (C)	136.1 (C)	37.7 (CH_2)	37.8 (CH_2)	21.7 (CH_3)	21.73 (CH_3)
134.6 (C)	134.6 (C)	35.3 (CH_2)	35.4 (CH_2)	21.6 (CH_3)	21.67 (CH_3)
118.0 (CH)	118.0 (CH)	34.6 (CH)	34.6 (CH)	14.4 (CH_3)	14.4 (CH_3)
52.1 (CH)	52.1 (CH)	27.0 (CH_2)	27.03 (CH_2)	13.6 (CH_3)	13.7 (CH_3)

[a] Blay, G.; Garcia, B.; Molina, E.; Pedro, J. R. *Org. Lett.* **2005**, *7*, 3291–3294. [b] 75 MHz, $CDCl_3$. [c] 150 MHz, $CDCl_3$.

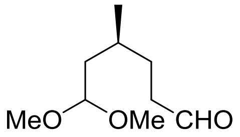

23

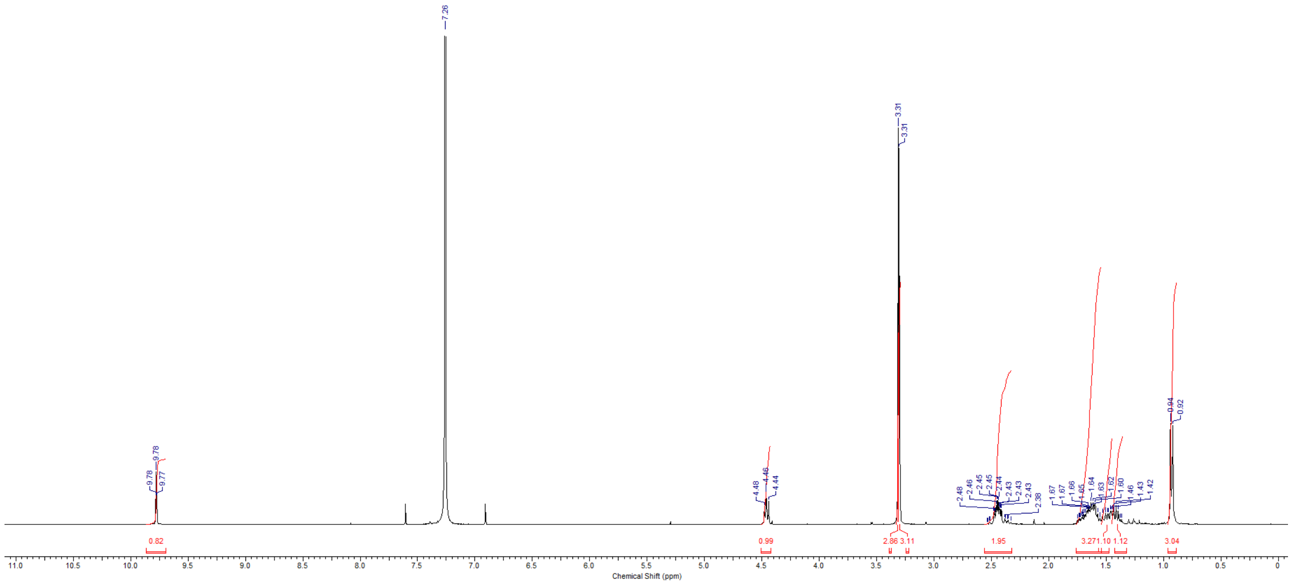

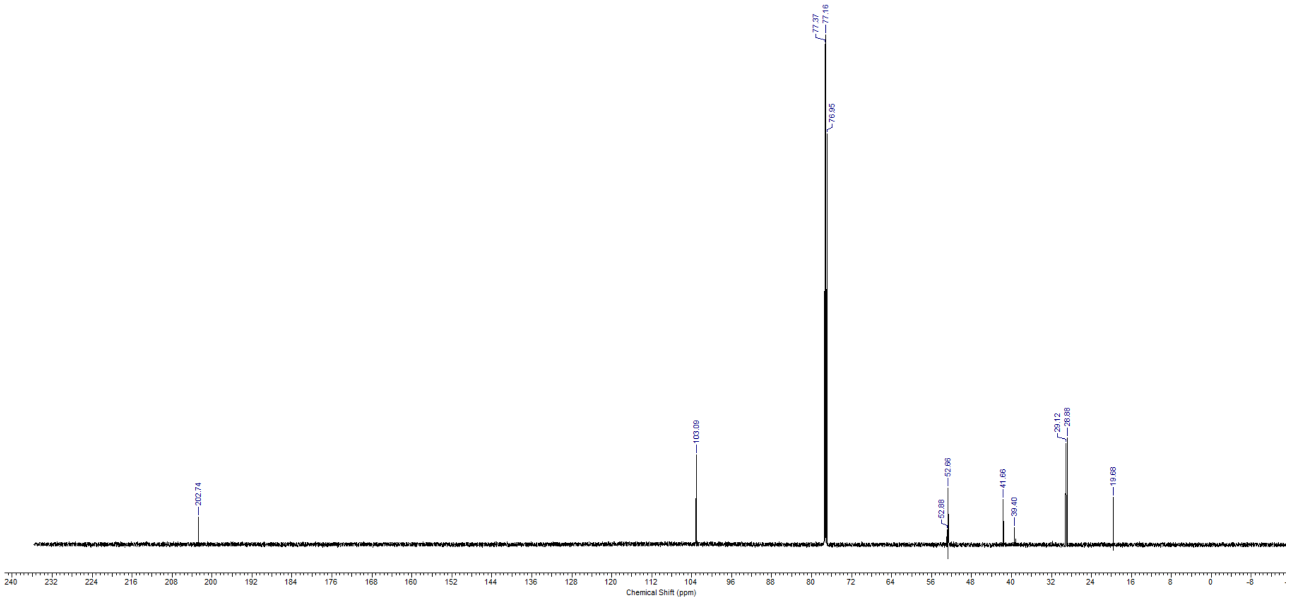

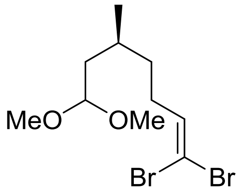

24

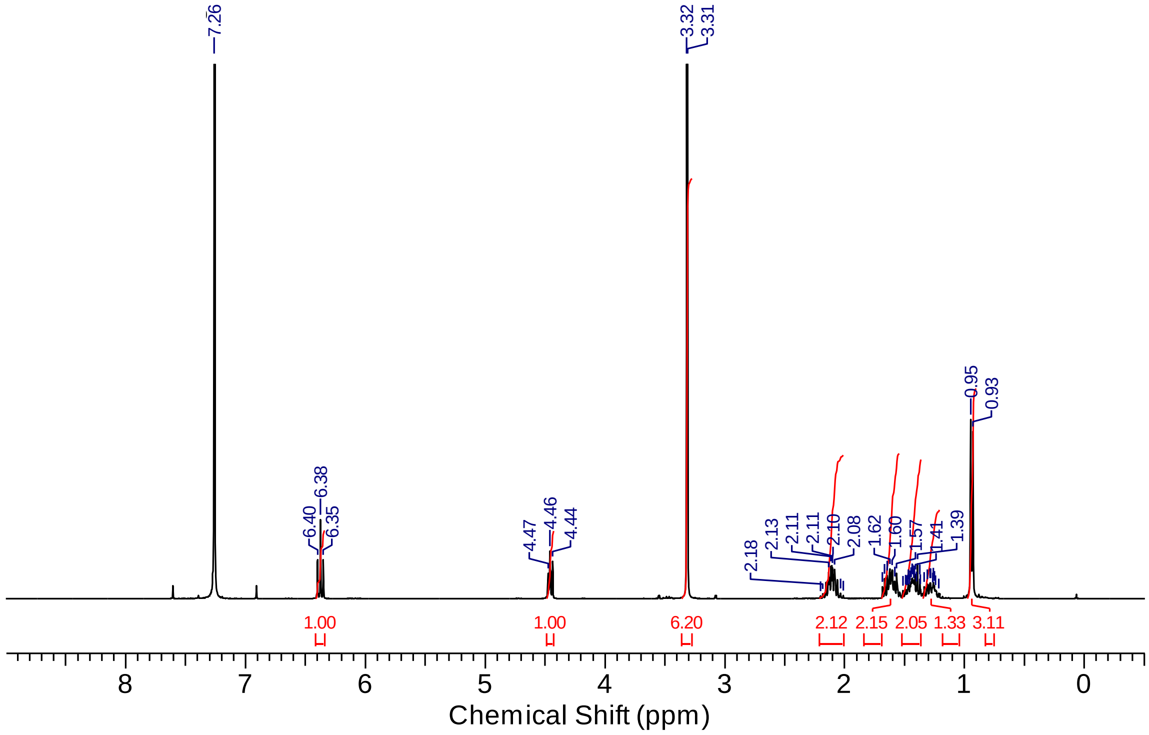

7.26
3.32
3.31
6.40
6.38
6.35
4.47
4.46
4.44
2.18
2.13
2.11
2.11
2.10
2.08
1.62
1.60
1.57
1.41
1.39
0.95
0.93
1.00
1.00
6.20
2.12
2.15
2.05
1.33
3.11
8
7
6
5
4
3
2
1
0
Chemical Shift (ppm)

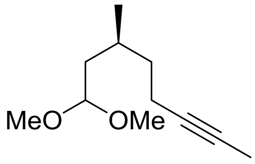
MeO
OMe

25

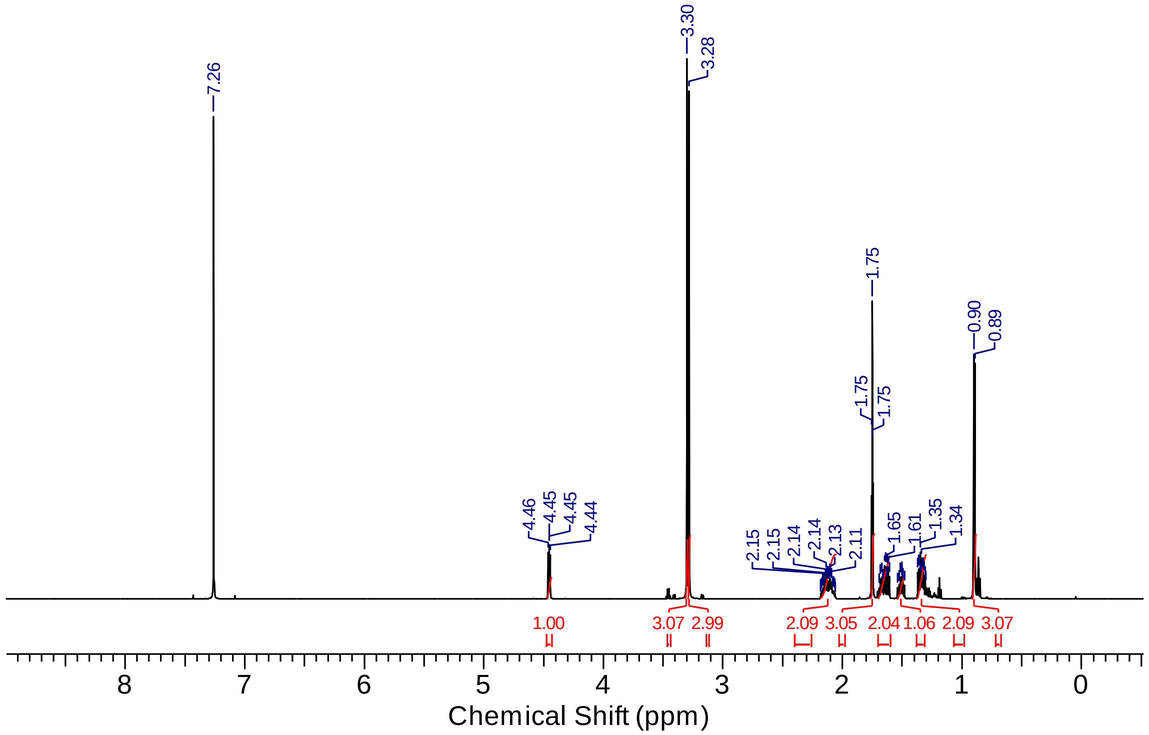
7.26
4.46
4.45
4.45
4.44
3.30
3.28
2.15
2.15
2.14
2.14
2.13
2.11
1.75
1.75
1.75
1.65
1.61
1.35
1.34
0.90
0.89
1.00
3.07
2.99
2.09
3.05
2.04
1.06
2.09
3.07
8
7
6
5
4
3
2
1
0
Chemical Shift (ppm)

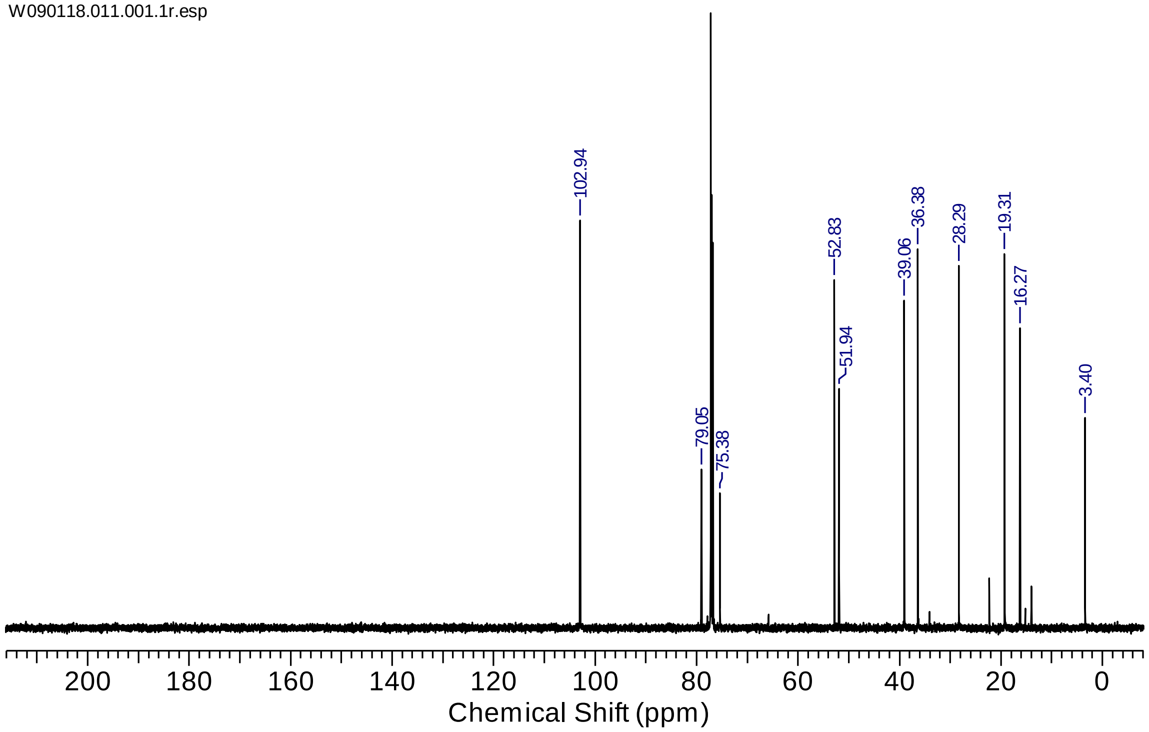

W090118.011.001.1r.esp
102.94
79.05
75.38
52.83
51.94
39.06
36.38
28.29
19.31
16.27
3.40
200
180
160
140
120
100
80
60
40
20
0
Chemical Shift (ppm)

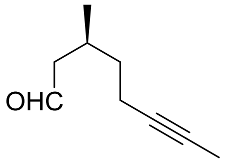

26

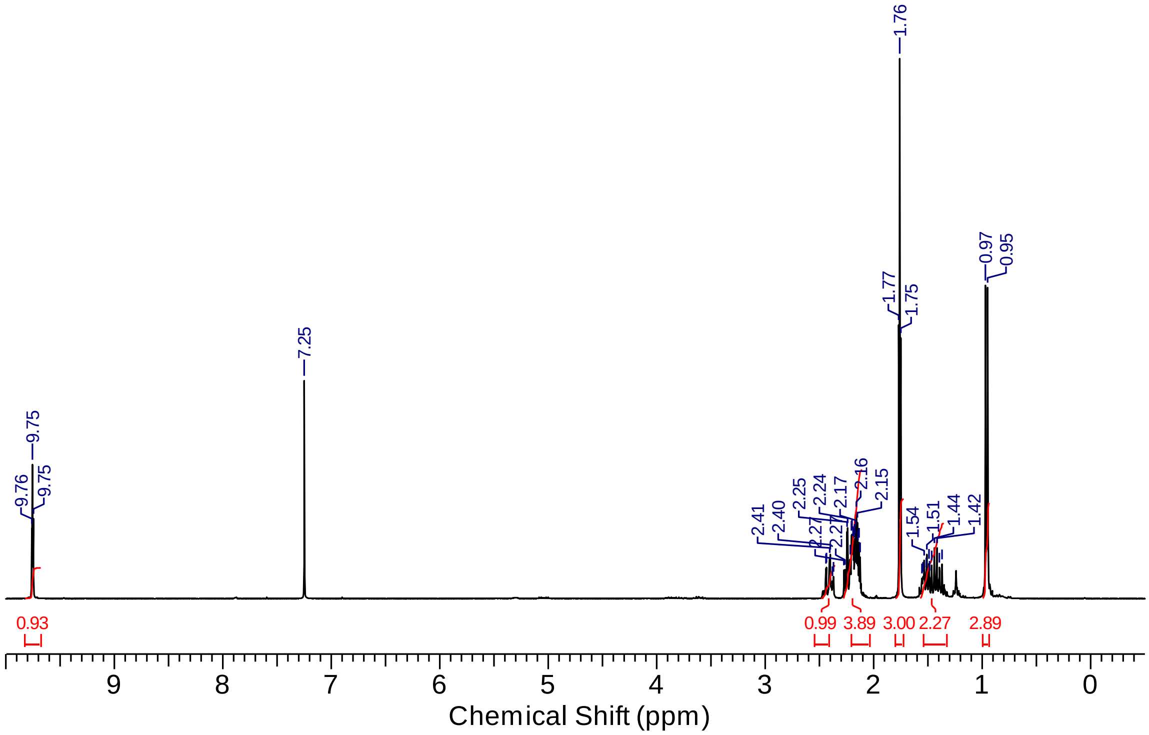

9.76
9.75
9.75
7.25
2.41
2.40
2.27
2.27
2.25
2.24
2.17
2.16
2.15
1.77
1.76
1.75
1.54
1.51
1.44
1.42
0.97
0.95
0.93
0.99
3.89
3.00
2.27
2.89
9
8
7
6
5
4
3
2
1
0
Chemical Shift (ppm)

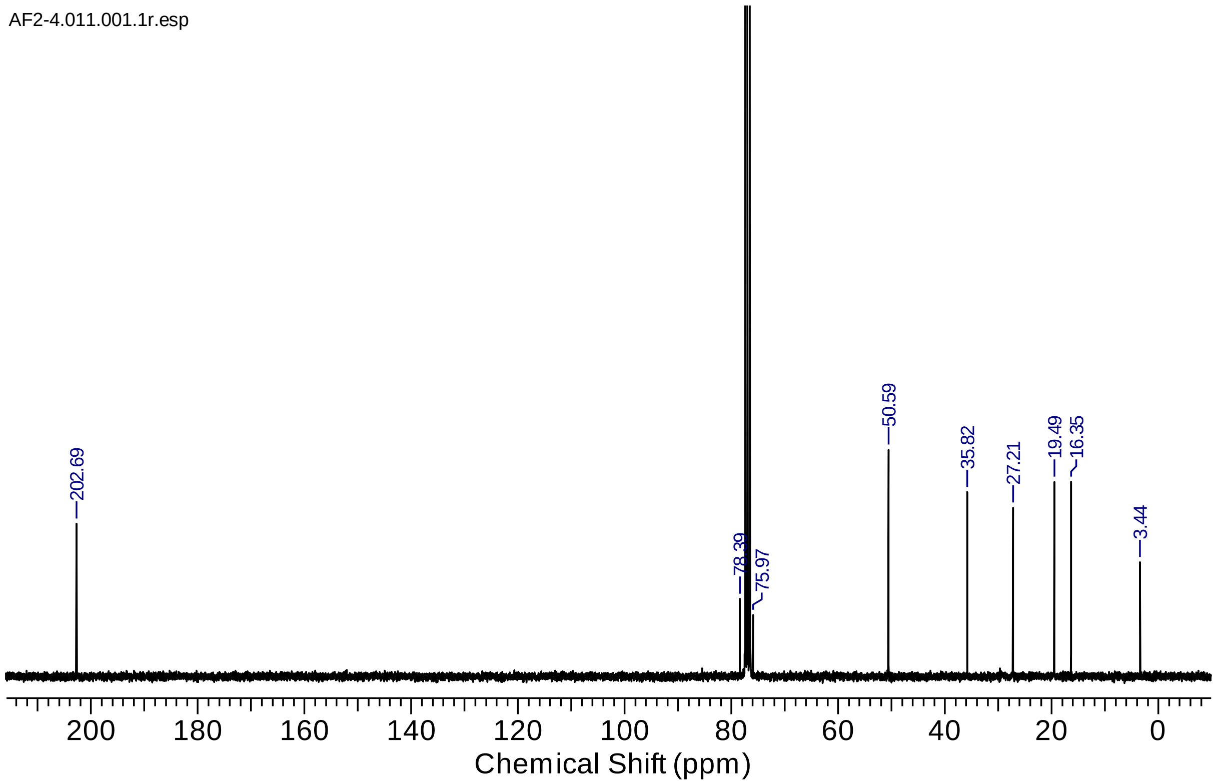
AF2-4.011.001.1r.esp
202.69
78.39
75.97
50.59
35.82
27.21
19.49
16.35
3.44
200
180
160
140
120
100
80
60
40
20
0
Chemical Shift (ppm)

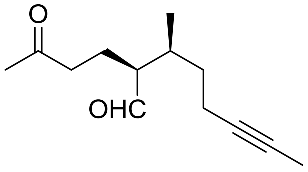

O
OHC

27

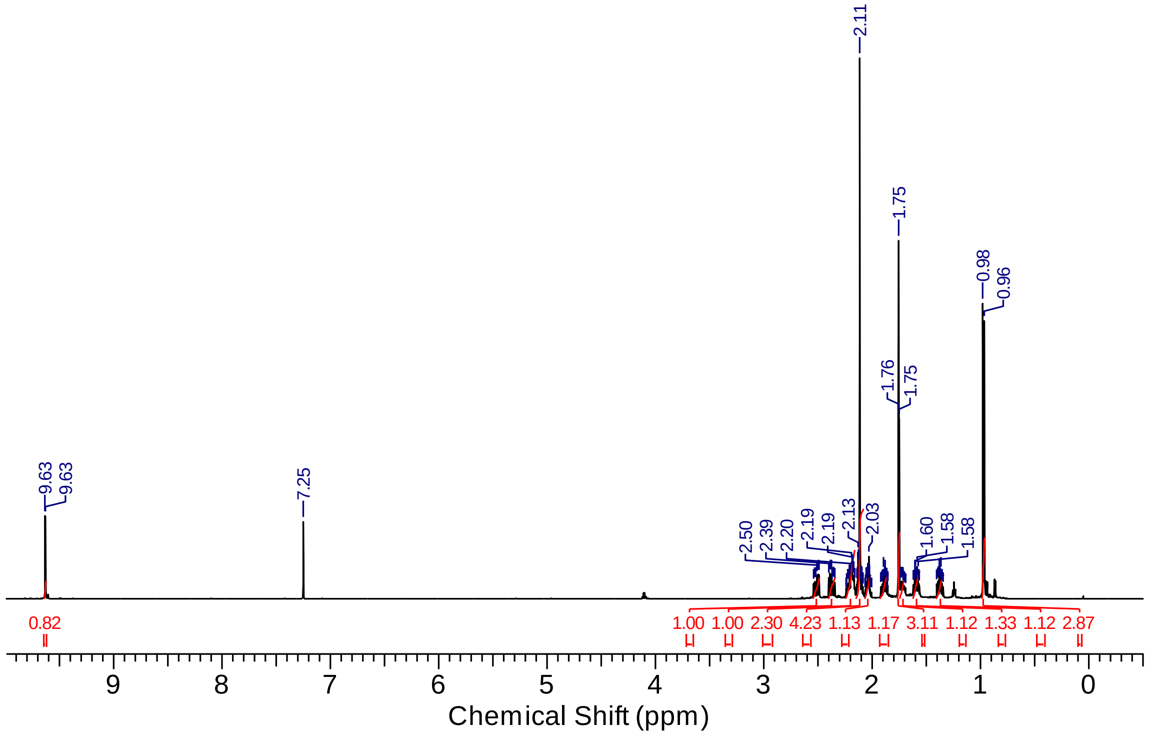

9.63
9.63
7.25
2.50
2.39
2.20
2.19
2.19
2.13
2.11
2.03
1.76
1.75
1.75
1.60
1.58
1.58
0.98
0.96
0.82
1.00 1.00 2.30 4.23 1.13 1.17 3.11 1.12 1.33 1.12 2.87
9 8 7 6 5 4 3 2 1 0
Chemical Shift (ppm)

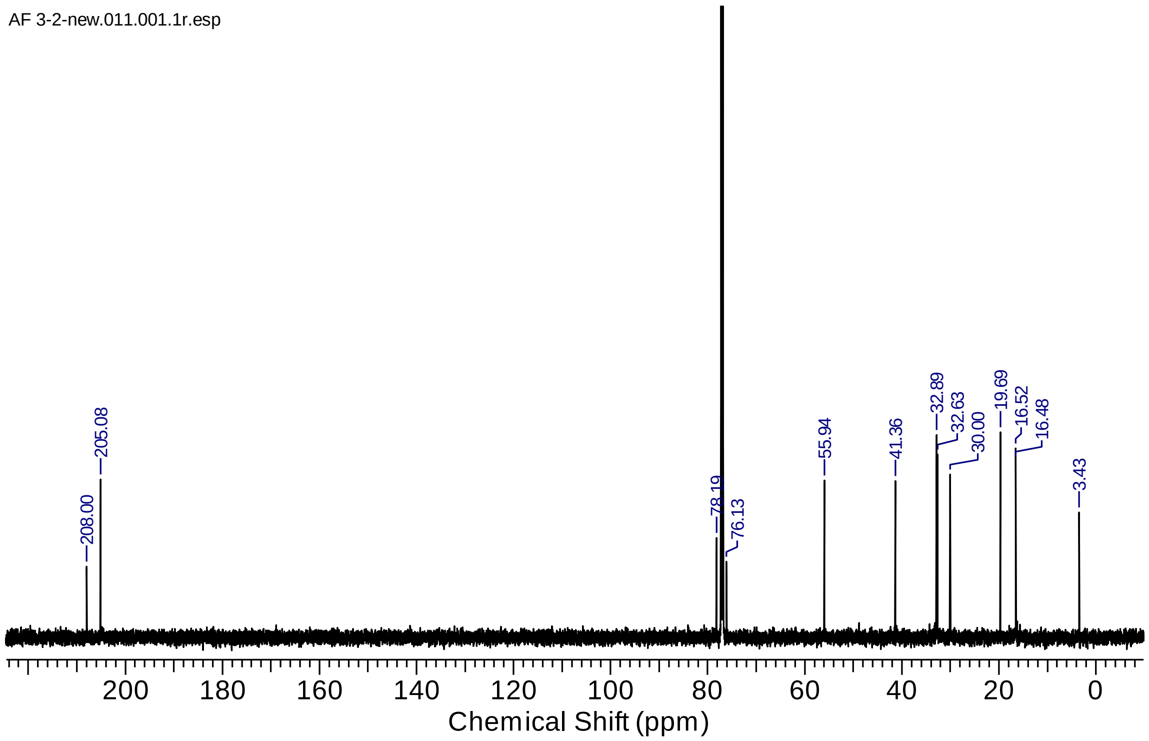
AF 3-2-new.011.001.1r.esp
208.00
205.08
78.19
76.13
55.94
41.36
32.89
32.63
30.00
19.69
16.52
16.48
3.43
200
180
160
140
120
100
80
60
40
20
0
Chemical Shift (ppm)

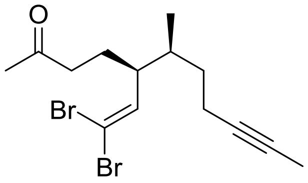
O
Br
Br

28

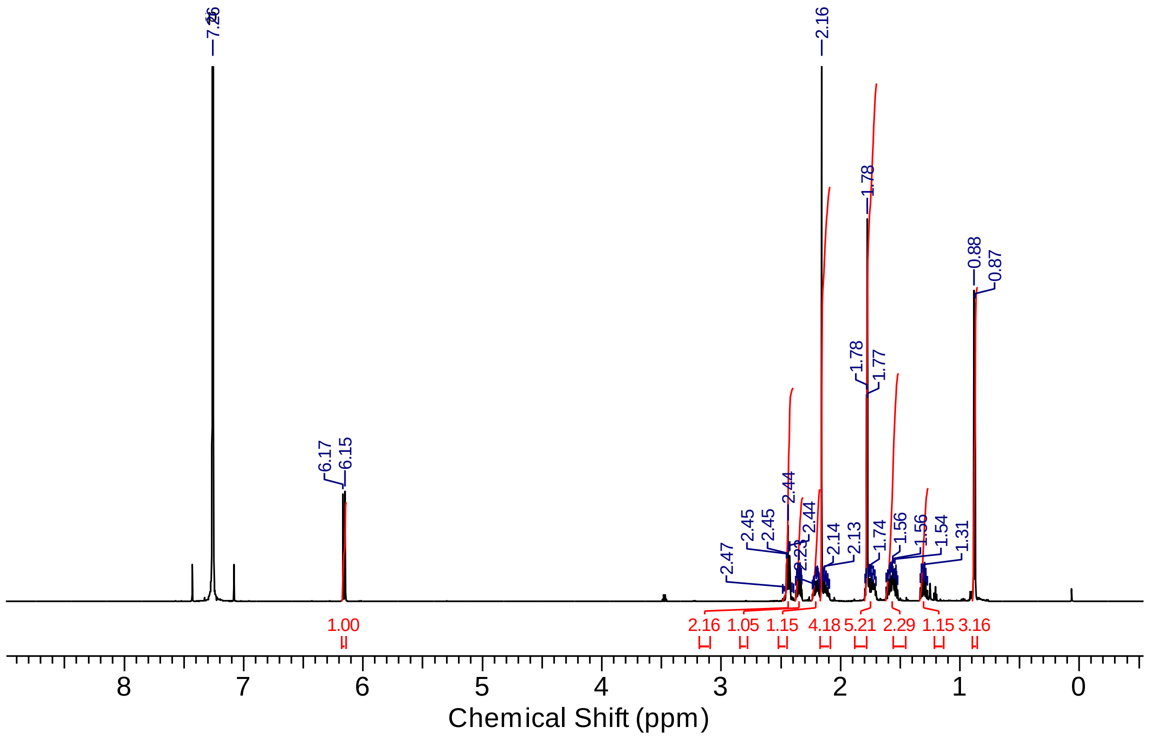
7.26
6.17
6.15
2.47
2.45
2.45
2.44
2.44
2.33
2.16
2.14
2.13
1.78
1.78
1.77
1.74
1.56
1.56
1.54
1.31
0.88
0.87
1.00
2.16
1.05
1.15
4.18
5.21
2.29
1.15
3.16
8
7
6
5
4
3
2
1
0
Chemical Shift (ppm)

2W310118.011.001.1r.esp

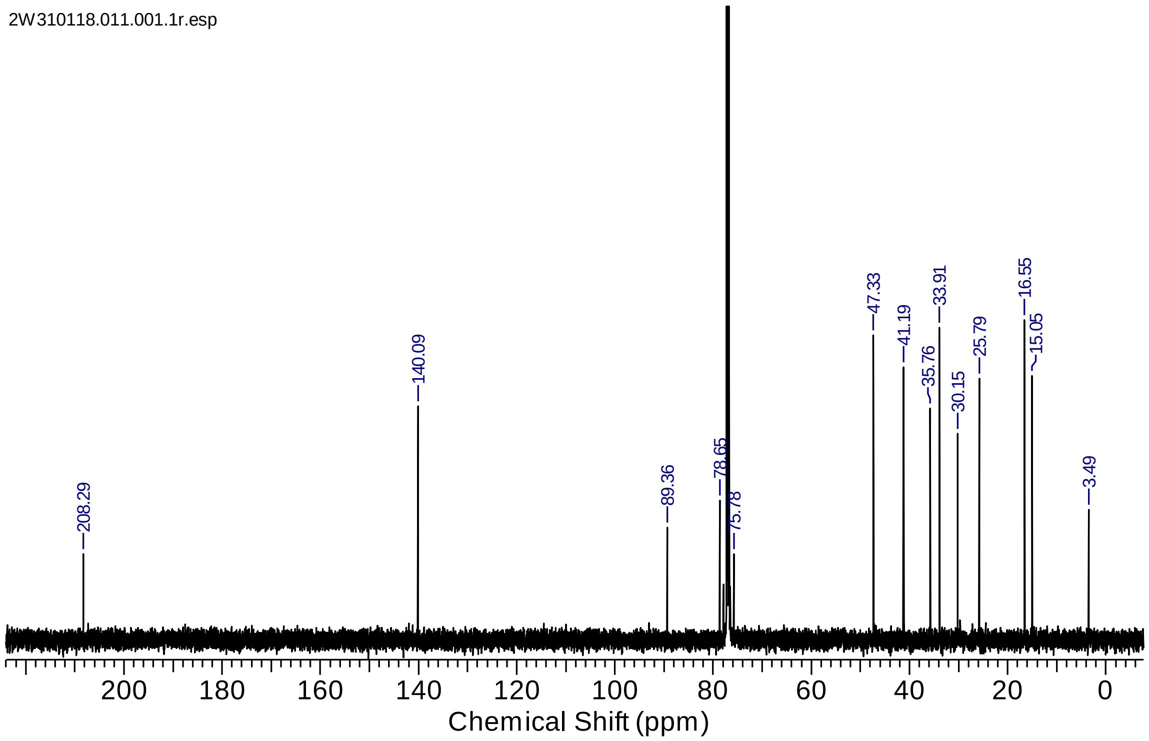

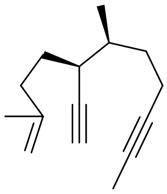

7

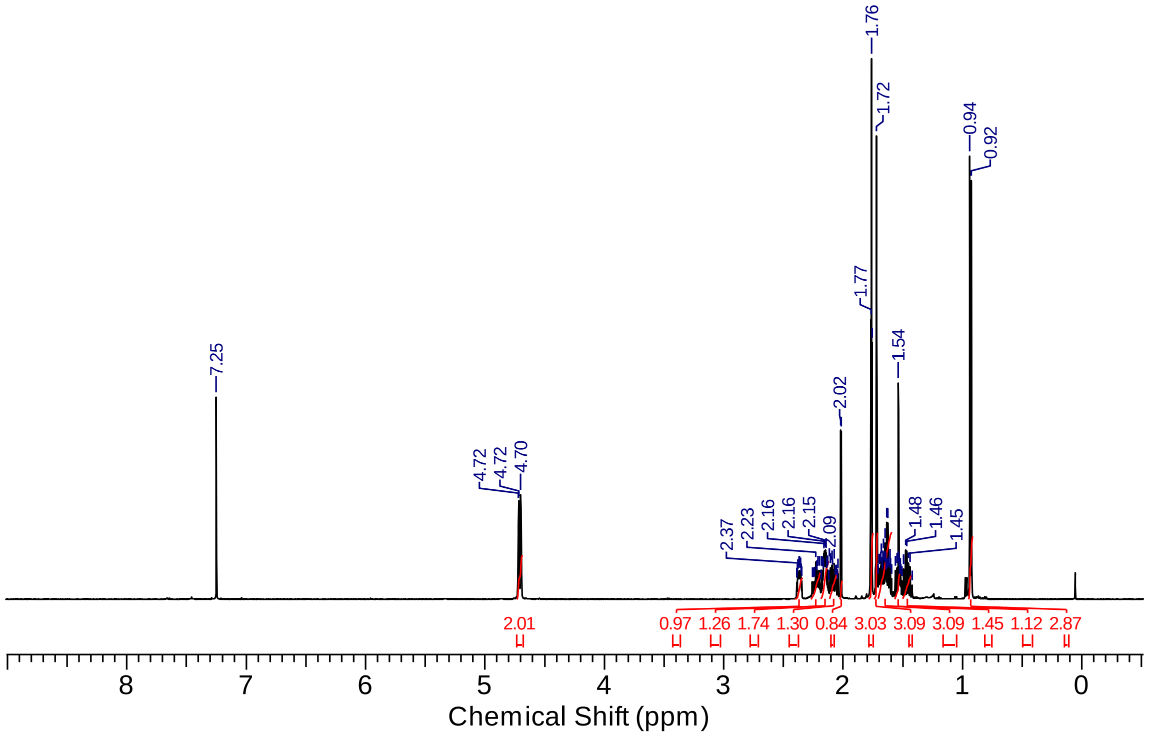

7.25
4.72
4.72
4.70
2.37
2.23
2.16
2.16
2.15
2.09
2.02
1.77
1.76
1.72
1.54
1.48
1.46
1.45
0.94
0.92
2.01
0.97 1.26 1.74 1.30 0.84 3.03 3.09 3.09 1.45 1.12 2.87
8 7 6 5 4 3 2 1 0
Chemical Shift (ppm)

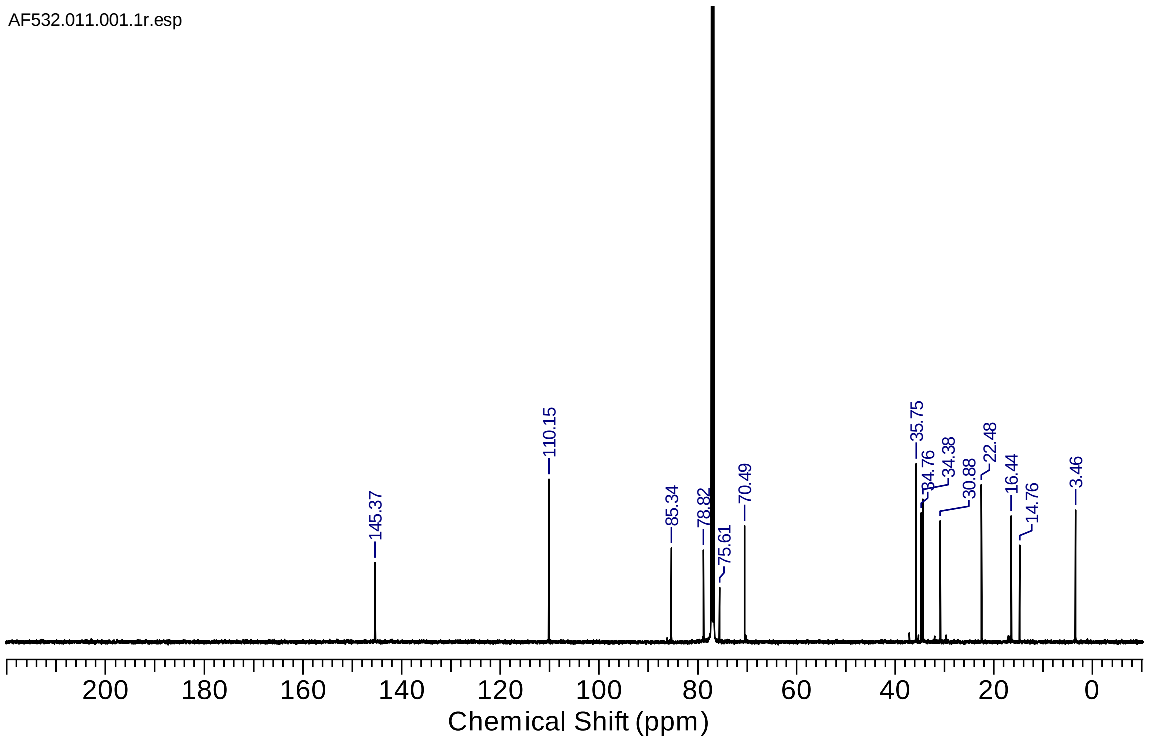

AF532.011.001.1r.esp
145.37
110.15
85.34
78.82
75.61
70.49
35.75
34.76
34.38
30.88
22.48
16.44
14.76
3.46
200
180
160
140
120
100
80
60
40
20
0
Chemical Shift (ppm)

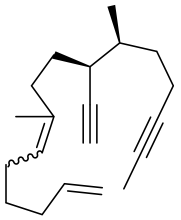

8

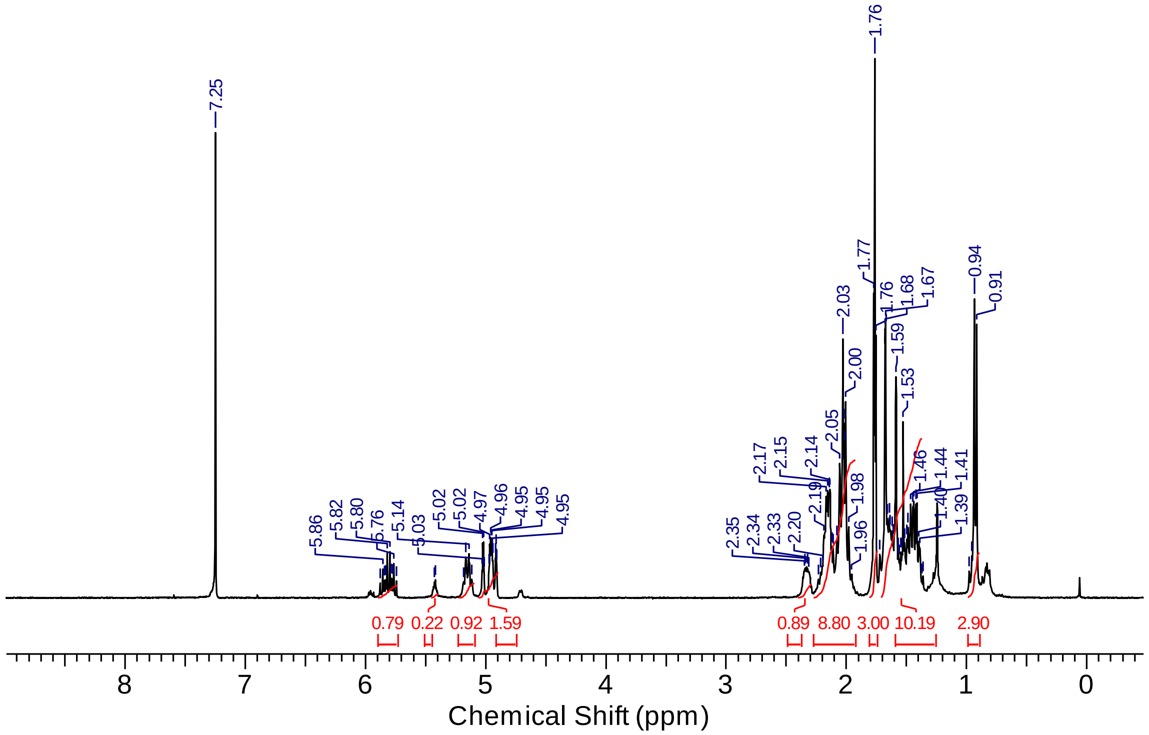

7.25
1.76
5.86
5.82
5.80
5.76
5.14
5.03
5.02
5.02
4.97
4.96
4.95
4.95
4.95
2.35
2.34
2.33
2.20
2.17
2.15
2.14
2.19
2.05
2.03
2.00
1.98
1.96
1.77
1.76
1.68
1.67
1.59
1.53
1.46
1.44
1.40
1.41
1.39
0.94
0.91
0.79
0.22
0.92
1.59
0.89
8.80
3.00
10.19
2.90
8
7
6
5
4
3
2
1
0
Chemical Shift (ppm)

AF5-9-1.011.001.1r.esp

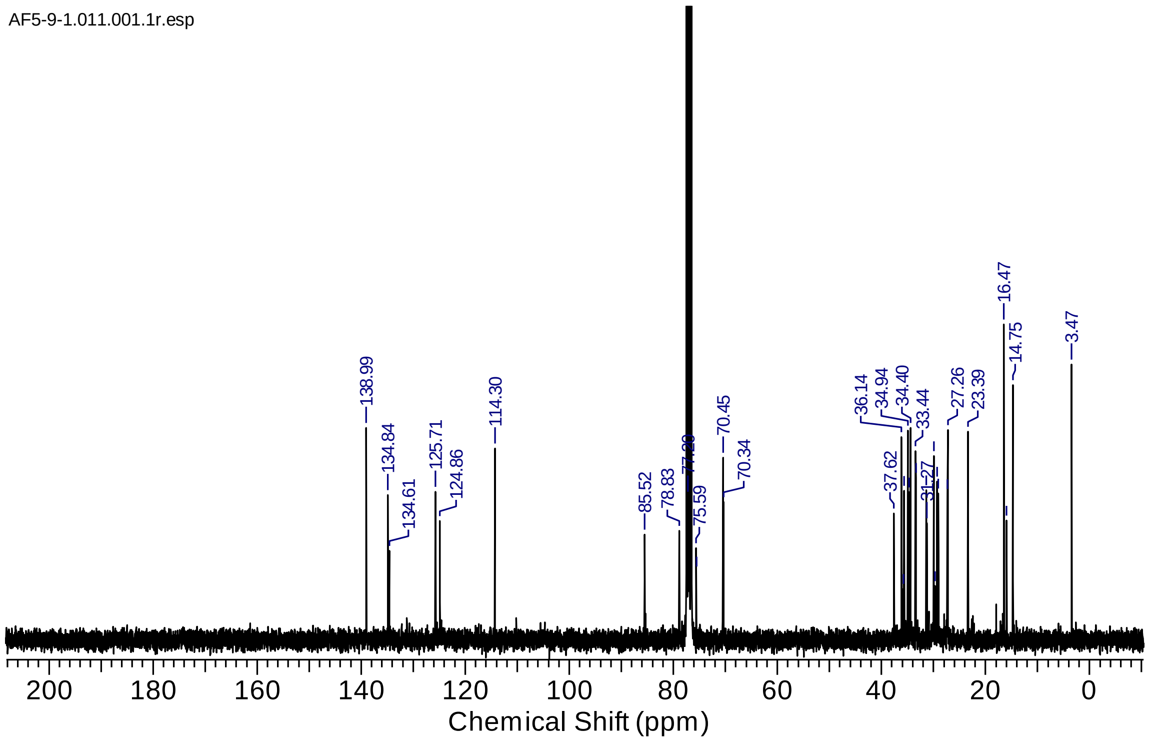

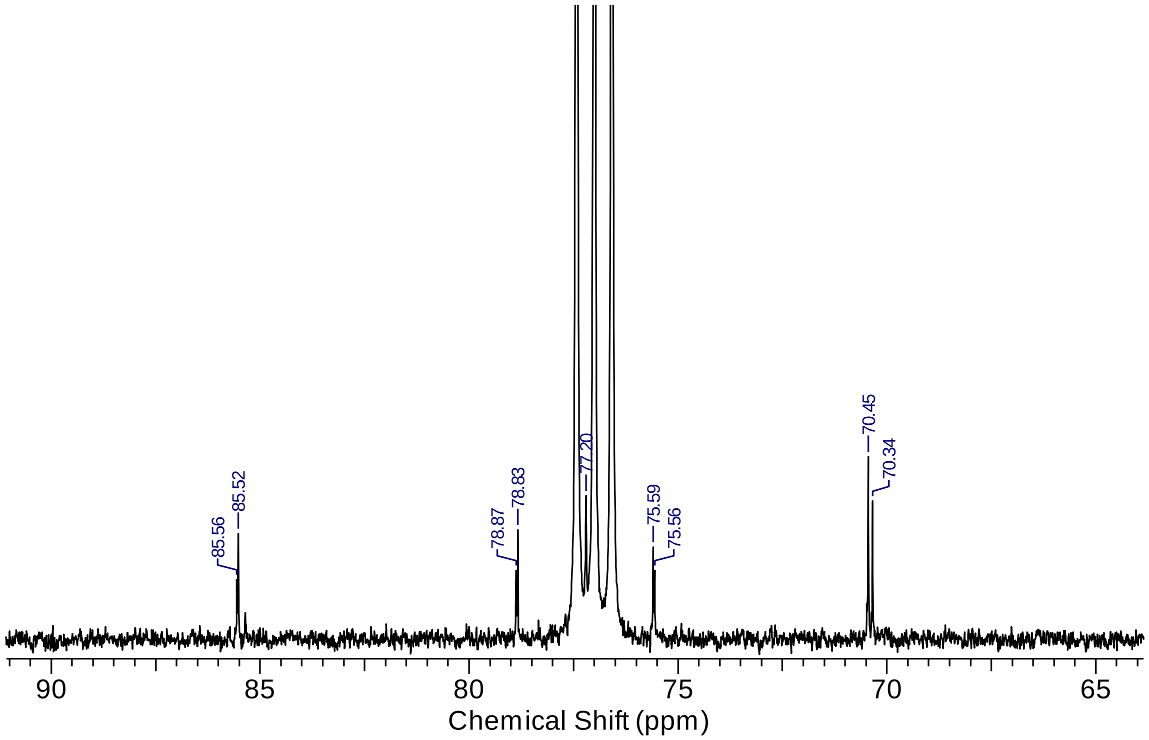

AF5-9-1.011.001.1r.esp

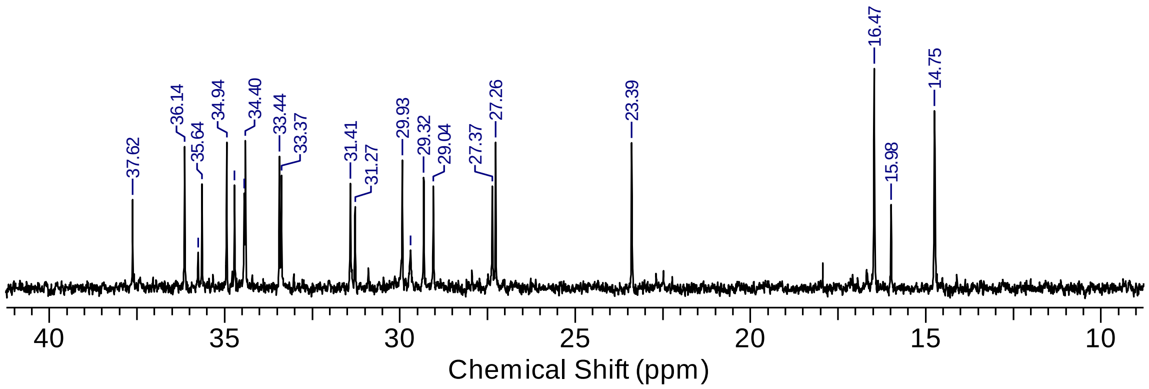

Supplement: File 1 — Experimental procedures and copies of 1H NMR and 13C NMR spectra of compounds 1, 3, 4, 7, 8, 10, 12, 14, 18, 20, 23–28. [file Beilstein_J_Org_Chem-15-858-s001.pdf]
